# Age-Dependent Activation of Purinergic Transmission Contributes to the Development of Epileptogenesis in ADSHE Model Rats

**DOI:** 10.3390/biom14020204

**Published:** 2024-02-08

**Authors:** Kouji Fukuyama, Eishi Motomura, Motohiro Okada

**Affiliations:** Department of Neuropsychiatry, Division of Neuroscience, Graduate School of Medicine, Mie University, Tsu 514-8507, Japan; k-fukuyama@clin.medic.mie-u.ac.jp (K.F.); motomura@clin.medic.mie-u.ac.jp (E.M.)

**Keywords:** ADSHE, astrocytes, ATP, epileptogenesis, P2X7 receptor

## Abstract

To explore the developmental processes of epileptogenesis/ictogenesis, this study determined age-dependent functional abnormalities associated with purinergic transmission in a genetic rat model (S286L-TG) of autosomal-dominant sleep-related hypermotor epilepsy (ADSHE). The age-dependent fluctuations in the release of ATP and L-glutamate in the orbitofrontal cortex (OFC) were determined using microdialysis and ultra-high-performance liquid chromatography with mass spectrometry (UHPLC-MS). ATP release from cultured astrocytes was also determined using UHPLC-MS. The expressions of P2X7 receptor (P2X7R), connexin 43, phosphorylated-Akt and phosphorylated-Erk were determined using capillary immunoblotting. No functional abnormalities associated with purinergic transmission could be detected in the OFC of 4-week-old S286L-TG and cultured S286L-TG astrocytes. However, P2X7R expression, as well as basal and P2X7R agonist-induced ATP releases, was enhanced in S286L-TG OFC in the critical ADSHE seizure onset period (7-week-old). Long-term exposure to a modest level of P2X7R agonist, which could not increase astroglial ATP release, for 14 d increased the expressions of P2X7R and connexin 43 and the signaling of Akt and Erk in astrocytes, and it enhanced the sensitivity of P2X7R to its agonists. Akt but not Erk increased P2X7R expression, whereas both Akt and Erk increased connexin 43 expression. Functional abnormalities, enhanced ATP release and P2X7R expression were already seen before the onset of ADSHE seizure in S286L-TG. Additionally, long-term exposure to the P2X7R agonist mimicked the functional abnormalities associated with purinergic transmission in astrocytes, similar to those in S286L-TG OFC. Therefore, these results suggest that long-term modestly enhanced purinergic transmission and/or activated P2X7R are, at least partially, involved in the development of the epileptogenesis of ADSHE, rather than that of ictogenesis.

## 1. Introduction

The traditional hypothesis regarding the imbalance between glutamatergic and GABAergic transmissions has contributed to the development of various antiseizure drugs (ASDs) [1,2,3,4,5]; however, the development of more effective ASDs, including those effective for ASD-resistant epilepsy, has remained a clinical challenge, because of the lack of a clear, detailed pathogenesis of epilepsy [2,6,7,8]. Accumulated preclinical findings have also suggested that dysfunctions other than glutamatergic/GABAergic transmissions contribute to epileptogenesis/ictogenesis [2,6,8,9,10]. Numerous mutations associated with autosomal-dominant sleep-related hypermotor epilepsy (ADSHE) have been identified in various genes, including coding nicotinic acetylcholine receptor α4 subunit (CHRNA4), from the ADSHE pedigrees [11,12,13,14,15,16,17,18,19,20,21,22,23,24,25,26,27,28,29,30,31,32,33,34,35,36]. ADSHE seizures are usually controlled by carbamazepine, while ADSHE patients with S284L-mutant CHRNA4 exhibit specific clinical characteristics, including resistance to carbamazepine and comorbid intellectual disability/autism [2,37,38].

We have already succeeded in generating a genetic ADSHE rat model, so-called “S286L-TG”, bearing the missense S286L mutation in rat Chrna4, which corresponds to the S284L mutation in human CHRNA4 [2,39,40]. Some pathomechanisms of S286L-TG have also been identified [2,39,40,41,42,43]. Initially, loss-of-function of the nicotinic acetylcholine receptor (nAChR) [44,45,46] leads to GABAergic disinhibition, resulting in the activation of glutamatergic neurons in the thalamus [2,39,40,42,43,47]. The hyperactivation of thalamic glutamatergic neurons propagates excitabilities associated with physiological sleep spindle bursts to the cortex and basal ganglia [40,41]. Accumulating propagated excitabilities accelerate tripartite synaptic transmission in the frontal cortex and basal ganglia through the activation of astroglial hemichannels [39,40,42,43]. Finally, synergic interactions among hyperactivated astroglial hemichannels, GABAergic disinhibition and sleep-related physiological high-frequency oscillations (HFOs) generate epileptogenic fast ripple bursts [41]. These findings suggest that functional abnormalities in neurotransmission and tripartite synaptic transmission play fundamental roles in the epileptogenesis/ictogenesis of ADSHE patients with the S284L mutation or S286L-TG.

Functional abnormalities in tripartite synaptic transmission seem to provide plausible explanations for the pathophysiology of carbamazepine resistance in ADSHE patients with the S284L mutation [2,39]. Recently, the P2X7 receptor (P2X7R) has attracted particular attention as part of an epilepsy pathomechanism, as ATP is a major gliotransmitter, and P2X7R is a non-desensitizing cation channel with less ATP sensitivity compared to other P2X receptor subtypes in the P2X receptor family [6,48,49]. Therefore, exploring the functional abnormalities of purinergic transmission may provide additional information on the ADSHE pathomechanisms of S286L-TG. Based on our hypothesis, we have determined the age-dependent development of functional abnormalities associated with purinergic transmission, such as ATP release and the expression of P2X7R, in S286L-TG rats.

## 2. Materials and Methods

### 2.1. Experimental Animals

All experimental procedures, including animal care and protocols for animal experiments, were approved by the Animal Research Ethics Committee of the Mie University School of Medicine (No. 24-37-R3, 7 March 2018) and performed in accordance with the ethical guidelines established by the Institutional Animal Care and Use Committee at Mie University, Japan, and the “Animal Research: Reporting of In Vivo Experiments” guidelines [50].

A total of 120 male rats—wild-type littermates (*n* = 60) and S286L-TG (*n* = 60) (Sprague Dawley strain background, SLC, Shizuoka, Japan)—as well as pregnant female wild-type (*n* = 6) and S286L-TG (*n* = 2) rats (Sprague Dawley rat background: SLC) were housed individually in cages and kept in air-conditioned rooms (temperature, 22 ± 2 °C) with a 12 h light/dark cycle, with ad libitum access to food and water. Neonatal (0–48 h of age: total *n* = 48) wild-type Sprague Dawley rats (*n* = 36) and S286L-TG rats (*n* = 12) were used for culturing astrocytes in vitro.

### 2.2. Chemical Agents

The 2′(3′)-O-(4-benzoylbenzoyl)adenosine-5′-triphosphate tri(triethylammonium) salt (BzATP: relatively potent rat P2X7R agonist) [51,52], 2-(phenylthio)-N-[[tetrahydro-4-(4-phenyl-1-piperazinyl)-2H-pyran-4-yl]methyl-3-pyridinecarboxamide (JNJ47965567: selective P2X7R antagonist) [53], TAT-Gap19 (Gap19: selective connexin 43 inhibitor) [54], carbenoxolone (CBX: non-selective astroglial hemichannel inhibitor) [55] and 10-[4′-(N,N-diethylamino)butyl]−2-chlorophenoxazine hydrochloride (10-DEBC: Akt inhibitor) were obtained from Funakoshi (Tokyo, Japan). 5-(2-Phenyl-pyrazolo[1,5-a]pyridin-3-yl)-1H-pyrazolo[3,4-c]pyridazin-3-ylamine (FR180204: Erk inhibitor) was obtained from the Tokyo Chemical Industry (Tokyo, Japan).

All compounds were prepared on the day of the experiment. JNJ47965567 was initially dissolved in 1 N HCl (50 mM). FR180204 was initially prepared as a 10 mM stock in dimethyl sulfoxide. BzATP, Gap19, CBX and 10-DEBC were dissolved directly in the experimental medium.

### 2.3. Preparation of the Microdialysis System

Rats were anesthetized with 1.8% isoflurane and placed on a stereotaxic frame. Concentric direct insertion-type dialysis probes (0.22 mm diameter, 3 mm exposed membrane: Eicom, Kyoto, Japan) were implanted in the orbitofrontal cortex (OFC: A = +3.2 mm, L = +2.4 mm, V = −6.5 mm, relative to bregma). Perfusion experiments began 18 h after recovery from anesthesia at a constant rate of 2 μL/min with modified Ringer solution (MRS: 145 Na^+^, 2.7 K^+^, 1.2 Ca^2+^, 1.0 Mg^2+^ and 154.4 Cl**^−^**, buffered at pH 7.4, with 2 mM phosphate buffer and 1.1 mM Tris buffer) [39,40].

Extracellular ATP was measured 8 h after starting perfusion. When the coefficients of the variation in ATP level reached less than 5% over 60 min (stabilization), pretreatment data were obtained over 60 min (pretreatment period). This was followed by local perfusion of MRS with or without (control) target agents (1 μM JNJ47965567 or 100 μM CBX) [53,56]. To determine the effects of the local perfusion of BzATP in the OFC for 20 min, the perfusion medium was then changed from MRS containing the target agents to MRS containing the same target agent with BzATP (range 1–100 μM) for 20 min (BzATP-evoked stimulation) [57]. The sampling of perfusates was performed between 9:00 and 15:00, since a previous study already demonstrated that extracellular ATP levels in the suprachiasmatic nuclei fluctuate according to circadian rhythm, with maximum levels observed during the latter half of the dark phase [58].

Previous studies revealed that the onset of ADSHE seizures in S286L-TG was at 8 weeks old, but no functional abnormalities could be detected in 4-week-old S286L-TG [2,39,41,47,59]. Therefore, according to these previous findings, to determine age-dependent fluctuations in basal and BzATP-evoked ATP releases, a dialysis probe was inserted into the OFC of 4-, 7- and 10-week-old wild type and S286L-TG.

Each dialysate was injected into an ultra-high-pressure liquid chromatograph equipped with a mass spectrometer (UHPLC-MS). The location of the dialysis probe was verified at the end of each experiment using 300 μm-thick brain tissue slices (Vibratome 1000; Technical Products International INC, St. Louis, MO, USA).

### 2.4. Primary Cultured Astrocytes

Cortical primary cultured astrocytes from wild-type and S286L-TG (0–48 h of age) rats were prepared according to previous studies [60]. The cerebral hemispheres were then removed using a dissection microscope. The brain was chopped into fine pieces using scissors and triturated using a micropipette. The suspension was filtered through a 70 µm nylon mesh (BD, Franklin Lakes, NJ, USA) and then centrifuged. The pellets were resuspended in Dulbecco’s Modified Eagle’s Medium (D6546; Sigma-Aldrich, St. Louis, MO, USA) containing 10% fetal calf serum (fDMEM). After 14 days of culture (DIV14), on DIV28, astrocytes were trypsinized and seeded directly on a translucent polyethylene terephthalate membrane (1.0 μm) in 24-well plates (BD, Franklin Lakes, NJ, USA) at a density of 100 cells/cm^2^ for the experiments. The fDMEM was changed twice a week between DIV14 and DIV28. To determine the effects of long-term exposure to BzATP, cultured astrocytes were incubated in fDMEM containing BzATP (concentration: 1 μM) for 14 days (DIV14-28). On DIV28, astrocytes were washed out using artificial cerebrospinal fluid (ACSF) (150 mM Na^+^, 3.0 mM K^+^, 1.4 mM Ca^2+^, 0.8 mM Mg^2+^ and 5.5 mM glucose adjusted to pH = 7.3 using 20 mM HEPES buffer) for the experiments.

To clarify the acute concentration-dependent effects of BzATP, cultured astrocytes, which were incubated in fDMEM without 1 μM BzATP from DIV14 to DIV28, were incubated in ACSF containing BzATP (1–100 μM) with or without 1 μM JNJ47965567 or 20 μM Gap19. To clarify the effects of long-term exposure to BzATP (1 μM), the cultured astrocytes were incubated in fDMEM containing 1 μM BzATP (1 μM BzATP did not affect the astroglial ATP release) from DIV14 to DIV28. After the washout using ACSF, cultured astrocytes were incubated in ACSF containing BzATP (concentration: 1–100 μM) with or without 1 μM JNJ47965567 or 20 μM Gap19.

It has been established that various types of molecules associated with ionic signaling are expressed in the plasma membrane of astrocytes [61,62]. Astroglial hemichannels containing connexin 43 and pannexin 1 are also activated by membrane depolarization [63]. Furthermore, the functions of P2X7R, hemichannels containing connexin 43 and pannexin 1, were detected by voltage clamp [64]. Based on these findings, to explore the effects of membrane depolarizations on astroglial hemichannel activity, the effects of artificial HFOs on astroglial ATP release were determined. The accumulation of HFOs, including either physiological ripple burst or epileptogenic fast ripple burst [41,65], has been reported to play an important role in the epileptogenesis of S286L-TG [2,39,47]. HFOs are physiological and pathological oscillatory activities that occur within a limited frequency band ranging from 80 to 500 Hz, which clearly stand out from the baseline and persist for at least four oscillation cycles [41,65]. HFOs are composed of two frequency ranges: a relatively slow physiological procognitive ripple burst (80–250 Hz, tens of milliseconds in duration) and an epileptogenic fast ripple burst (250–500 Hz, milliseconds in duration). The cultured astrocytes were activated by artificial ripple burst or fast ripple burst stimulations using a bus drive amplifier (SEG-3104MG; Miyuki Giken, Tokyo, Japan). Ripple burst and fast ripple burst stimulations were set at a square wave direct current pulse output with a magnitude of 300 mV/mm^2^ [66]. The ripple burst-evoked stimulation comprised 10 stimuli at 200 Hz and 10 bursts (50% duty cycle) at burst intervals of 100 milliseconds/s [41,66]. A set of fast ripple-evoked stimulations comprised 10 stimuli at 500 Hz and 10 bursts (50% duty cycle) at burst intervals of 40 milliseconds/s. These HFO-evoked stimulation patterns were regulated using LabChart v8.2 software (AD Instruments, Dunedin, New Zealand). The amounts of energization during the ripple burst- and fast ripple burst-evoked stimulations were set to be equal. To determine the interaction between long-term HFO-evoked stimulations and exposure to 1 μM BzATP on astroglial ATP release, cultured astrocytes were incubated in fDMEM with or without 1 μM BzATP for 14 day (DIV14-28) and received ripple-evoked or fast ripple-evoked stimulations for 3 d (DIV25-28) [41]. At DIV28, after washing with ACSF, the cultured astrocytes were incubated in ACSF with or without 1 μM JNJ47965567 or 20 μM Gap19 with ripple burst or fast ripple burst stimulation.

### 2.5. Determination of Levels of ATP and L-Glutamate

The ATP levels were analyzed using UHPLC-MS (Acquity UHPLC H-Class equipped with an Acquity SQ Detector; Waters, Milford, MA, USA). The samples (5 μL aliquots) were automatically injected into an autosampler (Acquity UHPLC Sample Manager FTN; Waters) and separated using a graphite carbon column (particle size: 3 μm, 150 × 2.1 mm, Hypercarb; Thermo), maintained at 450 μL/min at 40 °C. The UHPLC-MS procedure for determining ATP levels was as follows. A linear gradient elution program was used for more than 10 min with mobile phases A (1 mM ammonium acetate buffer, pH11) and B (100% acetonitrile). The nitrogen flow rates of the desolvation and cone were set at 750 and 5 L/h, respectively. The temperature for desolvation was set at 450 °C. The cone voltage for the measurement of ATP (*m*/*z* = 508.2) was 34 V.

Levels of L-glutamate were determined using UHPLC (PU-4185; Jasco, Tokyo, Japan) with fluorescence resonance energy transfer detection (FP-4020; Jasco), after dual derivatization with isobutyryl-L-cysteine and o-phthalaldehyde. Derivative reagent solutions were prepared by dissolving isobutyryl-L-cysteine (2 mg) and o-phthalaldehyde (2 mg) in 0.1 mL ethanol, followed by the addition of 0.9 mL sodium borate buffer (0.2 M, pH 9.0). Automated pre-column derivatives were prepared by drawing up a 5 μL aliquot of the sample, standard or blank solution and 5 μL of the derivative reagent solution and allowing the two to react in reaction vials for 5 min before injection. The derivatized samples (5 μL) were injected using an autosampler (AS-4150; Jasco). The analytical column (Triat C18, particle 1.8 µm, 50 × 2.1 mm; YMC, Kyoto, Japan) was maintained at 45 °C, with the flow rate set at 500 μL/min. A linear gradient elution program was performed over 10 min with mobile phases A (0.05 M citrate buffer, pH 5.0) and B (0.05 M citrate buffer containing 30% acetonitrile and 30% methanol, pH 3.5). The excitation/emission wavelengths of the fluorescence detector were set at 345/455 nm.

### 2.6. Capillary Immunoblotting

To analyze the expression of P2X7R, connexin 43, phosphorylated Akt (pAkt) and phosphorylated Erk (pErk), OFC and cultured astrocytes were extracted using a Minute Plasma Membrane Protein Isolation Kit (Invent Biotechnologies, Plymouth, MN, USA).

A capillary immunoblotting analysis was performed using Wes (ProteinSimple, Santa Clara, CA, USA) according to the ProteinSimple user manual. Total lysates or plasma membrane fractions were mixed with master mix (ProteinSimple) until the final concentration of 1 × sample buffer, 1 × fluorescent molecular weight marker and 40 mM dithiothreitol was obtained, which was then heated at 95 °C for 5 min. Samples, blocking reagent, primary antibodies, horseradish peroxidase (HRP)-conjugated secondary antibody, chemiluminescent substrate (SuperSignal West Femto; Thermo Fisher Scientific, Waltham, MA, USA), and separation and stacking matrices were also distributed into designated wells in a 25-well plate. After plate loading, separation electrophoresis and immunodetection were performed in a capillary system, which was fully automated. Capillary immunoblotting was performed at room temperature using the default settings of the instrument. The capillaries were first filled with a separation matrix, followed by a stacking matrix and a sample loading of approximately 40 nL. During electrophoresis, the proteins were separated based on molecular weight through stacking and separation matrices at 250 V for 40 min and then immobilized on the capillary wall using proprietary photoactivated capture chemistry. The matrices were then washed again. Next, the capillaries were incubated with a blocking reagent for 15 min and the target proteins were probed with primary antibodies, followed by incubation with HRP-conjugated secondary antibodies (anti-rabbit HRP-conjugated IgG, A00098, 10 μg/mL; GenScript, Piscataway, NJ, USA). Antibodies against GAPDH (NB300–322, 1:100; Novus Biologicals, Littleton, CO, USA), P2X7R (APR-004,10 μg/mL; Alomone, Jerusalem, Israel), connexin 43 (C6219, 1:100; Sigma-Aldrich), Erk (AF1576, 10 μg/mL; R&D Systems, Minneapolis, MN, USA), phosphorylated Erk (AF1018, 5 μg/mL; R&D Systems), Akt (AF1775, 1 μg/mL; R&D Systems) and phosphorylated Akt (AF877, 5 μg/mL; R&D Systems) were diluted in Immuno Shot Platinum (CosmoBio, Tokyo, Japan).

### 2.7. Data Analysis

All experiments were designed with groups containing equal numbers of animals (*n* = 6), without a formal power analysis, according to previous studies. All values have been expressed as the mean ± standard deviation (SD), and *p*-values of <0.05 (two-tailed) were considered statistically significant in all tests. The levels of drugs for administration were selected based on the values reported in previous studies. Where possible, we aimed to randomize and blind the data. To determine the levels of ATP, L-glutamate and protein, the sample order of the autosamplers was set using random number tables.

The time-dependent effects of the perfusion of 100 μM BzATP into the OFC on extracellular levels of ATP and L-glutamate in the OFC between wild-type and S286L-TG rats were analyzed by multivariate analysis of variance (MANOVA) with Scheffe’s post hoc test using BellCurve for Excel version 3.2 (Social Survey Research Information Co., Ltd., Tokyo, Japan). When the data did not violate the assumption of sphericity (*p* > 0.05), the F value of the MANOVA was analyzed using the assumed degrees of freedom of sphericity. When the assumption of sphericity was violated (*p* < 0.05), the F value was analyzed using Chi–Muller-corrected degrees of freedom. When the F value for the level/time factors of the MANOVA was significant, the data were analyzed using Scheffe’s post hoc test.

The concentration-dependent effects of perfusion with BzATP (ranging from 1 to 100 μM) in the OFC on extracellular ATP levels in the OFC between wild-type and S286L-TG rats were analyzed using two-way analysis of variance (ANOVA) with Scheffe’s post hoc test by BellCurve for Excel. The concentration-dependent effects of BzATP-evoked stimulation on astroglial ATP release were also analyzed using two-way ANOVA with Scheffe’s post hoc test using BellCurve for Excel. The expressions of P2X7R, connexin 43, pAkt and pErk were analyzed using one-way or two-way ANOVA with Scheffe’s post hoc test using BellCurve for Excel.

### 2.8. Nomenclature of Targets and Ligands

The key protein targets and ligands in this article are hyperlinked to their corresponding entries at http://www.guidetopharmacology.org (access 5 February 2024) and are permanently archived in the Concise Guide to PHARMACOLOGY 2021/2022 [67]. The data and statistical analyses complied with the recommendations of the British Journal of Pharmacology on experimental design and analysis in pharmacology.

## 3. Results

### 3.1. Purinergic Transmission in the OFC

#### 3.1.1. Comparison of Extracellular ATP Levels in the OFC between the Wild Type and S286L-TG

**Figure 1:**
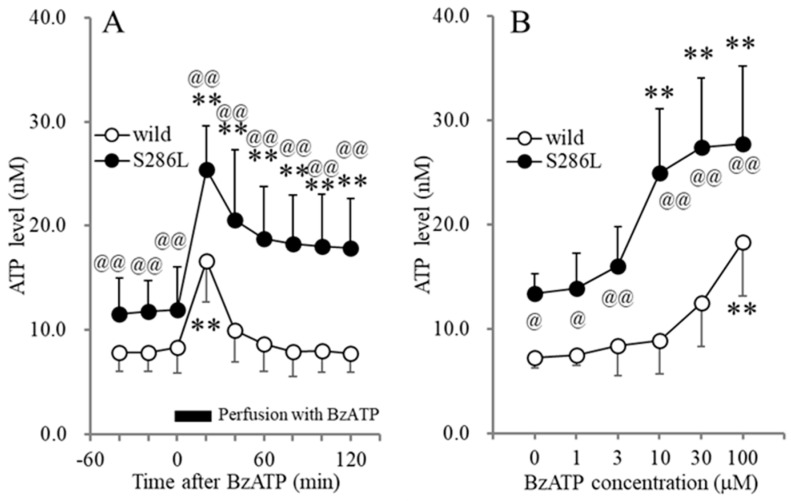
Effects of perfusion with BzATP into the OFC on extracellular ATP levels in the OFC of wild type and S286L-TG. Panel (**A**) indicates the time-dependent effects of perfusion with 100 μM BzATP into the OFC for 20 min (BzATP-evoked stimulation: black column) on extracellular ATP levels in the OFC of wild type (opened circles) and S286L-TG (closed circles) (7 weeks of age). The ordinate and abscissa indicate the mean ± SD (*n* = 6) of extracellular ATP levels (nM) and time after BzATP-evoked stimulation (min), respectively. ** *p* < 0.01 relative to pre-evoked stimulation (control) using MANOVA with Scheffe’s post hoc test [F_genotype_(1,10) = 18.3 (*p* < 0.01), F_BzATP_(6,60) = 61.3 (*p* < 0.01), F_genotype×BzATP_(6,60)= 7.5 (*p* < 0.01)]. Panel (**B**) indicates the concentration-dependent effects of perfusion with BzATP (ranging from 0 to 100 μM) into the OFC on extracellular ATP levels in the OFC of wild type (opened circles) and S286L-TG (closed circles). The ordinate and abscissa indicate the mean ± SD of extracellular ATP levels during BzATP perfusion (nM) and BzATP concentration (μM), respectively. ** *p* < 0.01 relative to BzATP free (0), @ *p* < 0.05, @@ *p* < 0.01 relative to the wild type using two-way ANOVA with Scheffe’s post hoc test [F_genotype_(1,60) = 95.2 (*p* < 0.01), F_BzATP_(5,60) = 17.4 (*p* < 0.01), F_genotype×BzATP_(5,60) = 3.0 (*p* < 0.05)].

The basal levels of extracellular ATP in the OFC of S286L-TG at the age of the onset of interictal discharges and before the onset of ADSHE onset (7-week-old) (11.6 ± 3.4 nM) were higher than those of wild type (7.9 ± 1.9 nM) (Figure 1). Perfusion with BzATP (1–100 μM) into the OFC increased extracellular ATP levels in the OFC (BzATP-evoked release) of both wild type and S286L-TG, in a concentration-dependent manner; however, the BzATP-evoked ATP release of S286L-TG was larger in comparison to that in the wild type (Figure 1). Perfusion with >10 μM BzATP increased ATP release in S286L-TG, while 100 μM BzATP was required to increase the ATP release in the wild type (Figure 1). After the discontinuation of 100 μM BzATP, the extracellular ATP levels of wild type immediately returned to baseline levels, while those of S286L-TG persisted, increasing the ATP release.

#### 3.1.2. Effects of JNJ47965567 and CBX on BzATP-Evoked ATP Release in the OFC of Wild-Type and S286L-TG Rats

To explore the mechanisms of discrepancies in BzATP-evoked ATP release between the wild type and S286L-TG, the effects of perfusion with 1 μM JNJ47965567 (selective P2X7R antagonist) [53,68] and 100 μM CBX (non-selective hemichannel inhibitor) [47,56,69,70,71,72,73,74] in the OFC on 100 μM BzATP-evoked ATP release in the OFC were determined.

**Figure 2:**
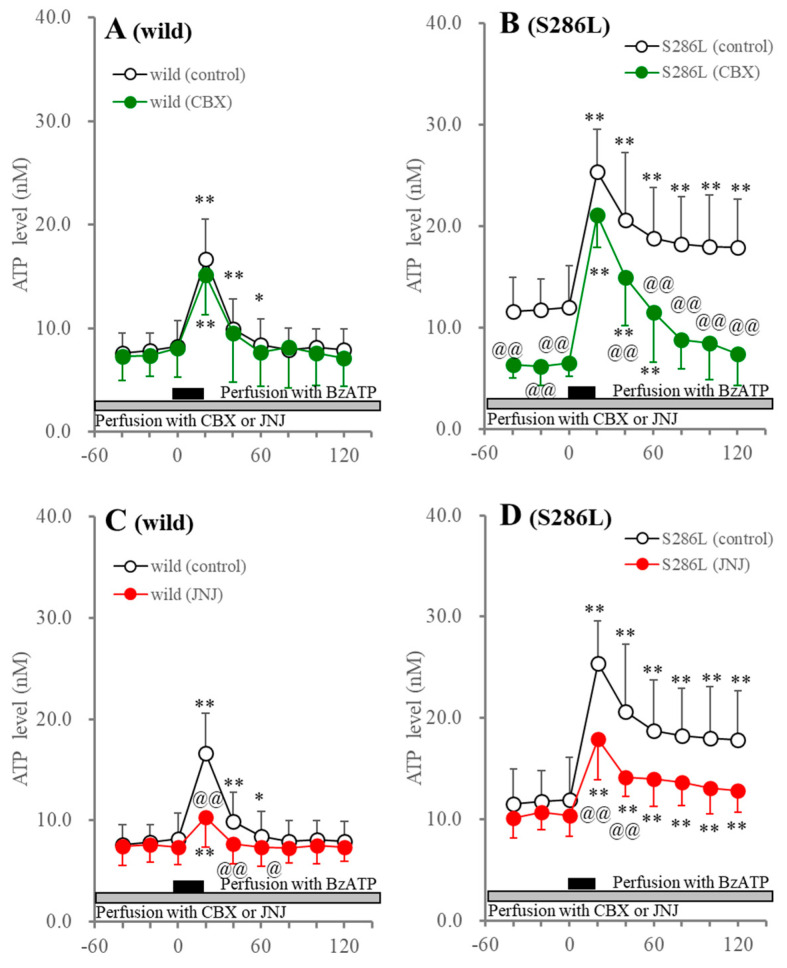
Effects of perfusion with carbenoxolone (CBX: panels (**A**,**B**)) and JNJ47965567 (JNJ: panels (**C**,**D**)) into the OFC on BzATP-evoked ATP release in the OFC of wild type (panels (**A**,**C**)) and S286L-TG (panels (**B**,**D**)). The ordinate and abscissa panels indicate the mean ± SD (*n* = 6) of the extracellular ATP levels (nM) and time after BzATP-evoked stimulation (min), respectively. Black and gray columns indicate perfusion with 100 μM BzATP and target agents (100 μM CBX or 1 μM JNJ47965567), respectively. * *p* < 0.05, ** *p* < 0.01, relative to before the BzATP-evoked stimulation, @ *p* < 0.05, @@ *p* < 0.01, relative to the control (BzATP alone) using MANOVA with Scheffe’s post hoc test. F values in effects of JNJ47965567 on BzATP-evoked ATP release of wild type and S286L-TG were [F_JNJ_(1,10) = 1.8 (*p* > 0.1), F_BzATP_(4.5,44.8) = 84.7 (*p* < 0.01), F_JNJ×BzATP_(4.5,44.8) = 4.3 (*p* < 0.01)] and [F_JNJ_(1,10) = 1.1 (*p* > 0.1), F_BzATP_(5.5,54.8) = 32.4 (*p* < 0.01), F_JNJ×BzATP_(5.5,54.8) = 10.0 (*p* < 0.01)], respectively. F values in effects of CBX on BzATP-evoked ATP release of wild type and S286L-TG were [F_CBX_(1,10) = 0.1 (*p* > 0.1), F_BzATP_(3.8,38.3) = 65.2 (*p* < 0.01), F_CBX×BzATP_(3.8,38.3) = 0.5 (*p* > 0.1)] and [F_CBX_(1,10) = 10.5 (*p* < 0.01), F_BzATP_(6,60) = 77.3 (*p* < 0.01), F_CBX×BzATP_(6,60) = 5.5 (*p* < 0.01)], respectively.

Perfusion with 1 μM JNJ47965567 suppressed BzATP-evoked ATP release, but did not affect the basal ATP release of both wild type and S286L-TG. Importantly, persistently increasing ATP release after BzATP discontinuation (40–120 min) in S286L-TG was yet to be observed (Figure 2). Therefore, BzATP-evoked ATP release is mainly generated by the activation of P2X7R in both the wild type and S286L-TG, whereas persistently increasing ATP release after BzATP discontinuation is probably induced not only by the activation of P2X7R, but also by other systems than P2X7R in S286L-TG.

It has been established that P2X7R forms a complex with pannexin 1, which is inhibited by CBX and contributes to the release of astroglial transmitters [75]. The hyperactivation of the astroglial hemichannels plays an important role in epileptogenesis/ictogenesis in S286L-TG [2,39,41,47]. Taking into account these previous findings, the increasing basal ATP release and the persistently increasing ATP release after the BzATP discontinuation in S286L-TG may reflect enhanced astroglial hemichannel function or the P2X7R/pannexin 1 complex in S286L-TG. Therefore, the effects of perfusion with 100 μM CBX on BzATP-induced ATP release were determined.

Perfusion with 100 μM CBX into the OFC did not affect basal or BzATP-evoked ATP release in the wild type; however, CBX decreased basal and persistently increased ATP release after BzATP discontinuation in S286L-TG, but did not affect them in the wild type (Figure 2). These discrepancies in basal and persistent ATP release after BzATP discontinuation between the wild type and S286L-TG indicate that the release of ATP in S286L-TG is probably regulated by the astroglial hemichannel and/or the P2X7R/pannexin 1 complex, while that of wild type is not affected.

#### 3.1.3. Effects of JNJ47965567 and CBX on BzATP-Evoked L-Glutamate Release in the OFC of Wild-Type and S286L-TG Rats

The persistent increase in BzATP-evoked ATP release in S286L-TG suggests the possibility of impaired ATP degradation in the extracellular space, such as reduced ecto-ATPase activity. However, persistently increasing extracellular L-glutamate levels in S286L-TG, through astroglial hemichannels, were also observed in several previous studies [39,40,42,43,47]. Additionally, both P2X7R and hemichannels are non-selectively permeable molecules, such as L-glutamate [2,48]. Therefore, based on these preclinical findings, to clarify the functional abnormalities of P2X7R, as well as the hemichannel and ATP degradation in the extracellular space in S286L-TG, the effects of perfusion with 1 μM JNJ47965567 and 100 μM CBX on 100 μM BzATP-evoked L-glutamate release in the OFC of wild type and S286L-TG were also determined.

**Figure 3:**
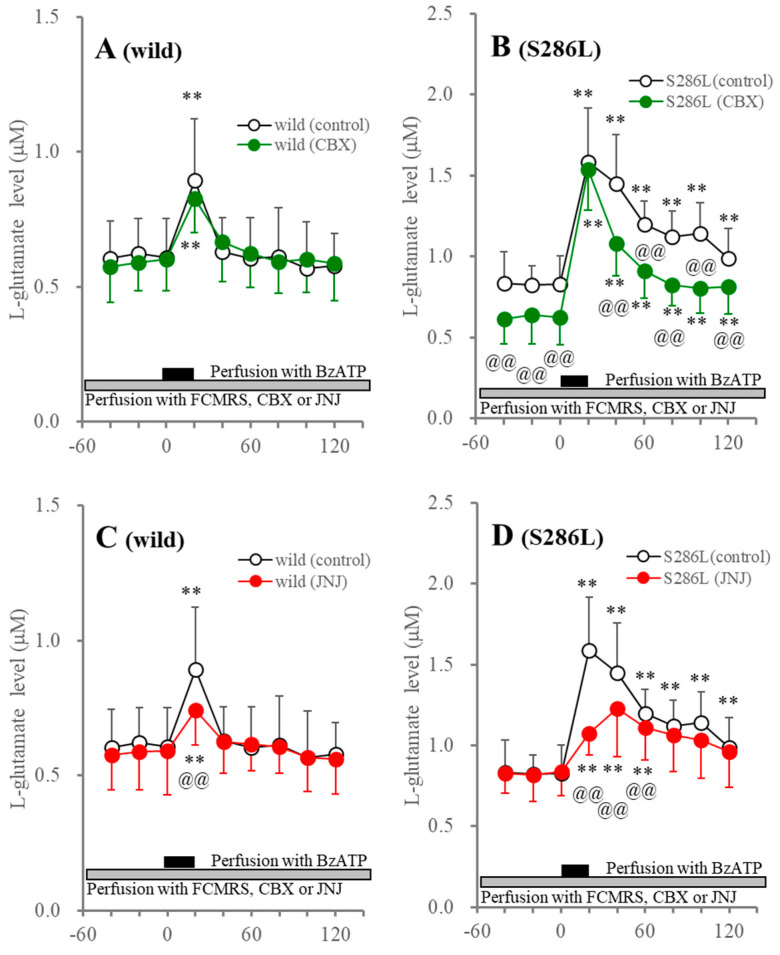
Effects of perfusion with CBX (panels (**A**,**B**)) and JNJ47965567 (JNJ: panels (**C**,**D**)) into the OFC on BzATP-evoked L-glutamate release in the OFC of wild type (panels (**A**,**C**)) and S286L-TG (panels (**C**,**D**)). The ordinate and abscissa indicate the mean ± SD (*n* = 6) of the extracellular L-glutamate levels (μM) and time after BzATP-evoked stimulation (min), respectively. Black and gray columns indicate perfusion with 100 μM BzATP and target agents (100 μM CBX or 1 μM JNJ47965567), respectively. ** *p* < 0.01, relative to before the BzATP-evoked stimulation, @@ *p* < 0.01, relative to control (BzATP alone) using MANOVA with Scheffe’s post hoc test. F values in effects of JNJ47965567 on BzATP-evoked L-glutamate release of wild type and S286L-TG were [F_JNJ_(1,10) = 0.1 (*p* > 0.1), F_BzATP_(6,60) = 34.6 (*p* < 0.01), F_JNJ×BzATP_(6,60) = 3.7 (*p* < 0.01)] and [F_JNJ_(1,10) = 1.5 (*p* > 0.1), F_BzATP_(3.3,33.3) = 51.3 (*p* < 0.01), F_JNJ×BzATP_(3.3,33.3) = 11.7 (*p* < 0.01)], respectively. F values in effects of CBX on BzATP-evoked ATP release of wild type and S286L-TG were [F_CBX_(1,10) = 0.1 (*p* > 0.1), F_BzATP_(3.4,34.1) = 48.2 (*p* < 0.01), F_CBX×BzATP_(3.4,34.1) = 1.7 (*p* > 0.1)] and [F_CBX_(1,10) = 5.0 (*p* < 0.05), F_BzATP_(2.7,27.3) = 145.4 (*p* < 0.01), F_CBX×BzATP_(2.7,27.3) = 6.0 (*p* < 0.01)], respectively.

Perfusion with JNJ47965567 suppressed the BzATP-evoked L-glutamate release without affecting the basal L-glutamate release in both the wild type and S286L-TG. After BzATP discontinuation, the L-glutamate level in the wild type immediately recovered to baseline levels, while the increasing L-glutamate release in S286L-TG was persistent (Figure 3). Perfusion with CBX did not affect basal or BzATP-evoked L-glutamate release in the wild type, but decreased the basal and persistently increasing L-glutamate release after BzATP discontinuation, without affecting the BzATP-evoked L-glutamate release in S286L-TG (Figure 3).

The similar pharmacological features of the release of ATP and L-glutamate suggest that the enhanced functions of the P2X7R and/or the astroglial hemichannel in S286L-TG, in comparison to the wild type, play important roles in the release of ATP and L-glutamate in the OFC. Additionally, persistent increases in BzATP-evoked L-glutamate release were also observed, similar to ATP release. Therefore, the similarity between BzATP-evoked ATP and L-glutamate releases suggests that the persistently increasing extracellular ATP levels are probably caused by enhanced astroglial releases through hemichannels, or P2X7R rather than impaired ATP degradation in the extracellular space.

#### 3.1.4. Age-Dependent Fluctuations in Extracellular ATP Levels in the OFC of Wild Type and S286L-TG

Functional abnormalities in transmission in S286L-TG were clearly detected and were critical in the period around the onset of ADSHE seizures (approximately 8 weeks old), but were not observed at 4 weeks old [2,39,47,59]. Therefore, to explore whether the enhanced BzATP-evoked ATP release in the OFC of S286L-TG is generated by age-dependent processes, most likely tripartite glutamatergic transmission, the BzATP-evoked ATP release was determined in the 4-, 7- and 10-week-old wild type and S286L-TG.

**Figure 4:**
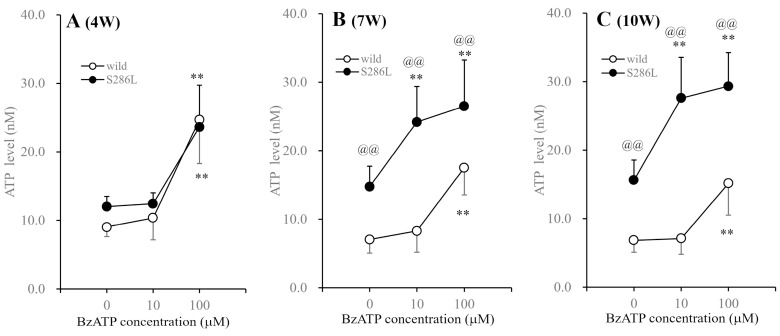
Age-dependent fluctuations in BzATP-evoked ATP releases in the OFC of wild type and S286L-TG. Panels (**A**–**C**) indicate the BzATP-evoked ATP releases of wild type (opened circles) and S286L-TG (closed circles) at 4, 7 and 10 weeks of age, respectively. Ordinates and abscissas indicate the mean ± SD (*n* = 6) of the extracellular ATP levels in the OFC (nM) and BzATP levels (μM), respectively. ** *p* < 0.01 relative to BzATP free (0), @@ *p* < 0.01 relative to the wild type using two-way ANOVA with Scheffe’s post hoc test. F values for 4, 7 and 10 weeks of age were [F_genotype_(1,30) = 1.0 (*p* > 0.1), F_BzATP_(2,30) = 44.4 (*p* < 0.01), F_genotype×BzATP_(2,30) = 0.8 (*p* > 0.1)], [F_genotype_(1,30) = 71.4 (*p* < 0.01), F_BzATP_(2,30) = 22.8 (*p* < 0.01), F_genotype×BzATP_(2,30) = 3.4 (*p* < 0.05)] and [F_genotype_(1,30) = 40.4 (*p* < 0.01), F_BzATP_(2,30) = 6.3 (*p* < 0.01), F_genotype×BzATP_(2,30) = 3.7 (*p* < 0.05)], respectively.

At 4 weeks old, the basal ATP release in the OFC of S286L-TG was almost equal to that of wild type, but increased at 7 and 10 weeks old compared to those of the wild type. At 4 weeks old, extracellular ATP levels in both the wild type and S286L-TG were increased by perfusion with 100 µM BzATP, but were not affected by 10 µM BzATP. Furthermore, the extracellular ATP levels in the wild type at 7 and 10 weeks old were not affected by 10 µM BzATP, while 100 µM BzATP increased ATP release. Contrarily, the extracellular ATP levels in S286L-TG were increased by 10 and 100 μM BzATP at both 7 and 10 weeks old (Figure 4).

#### 3.1.5. Age-Dependent Expression of P2X7R on the Plasma Membrane in the OFC of Wild-Type and S286L-TG Rats

**Figure 5:**
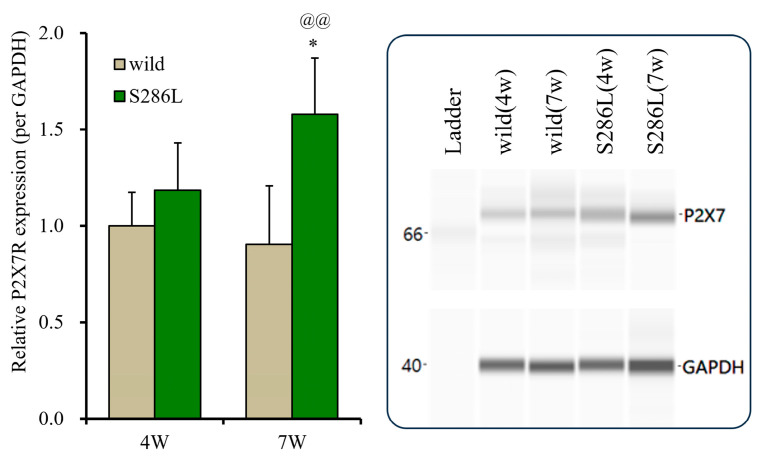
Age-dependent fluctuations in P2X7R expression on the plasma membrane in the OFC of wild type and S286L-TG. The left side panel indicates expression of P2X7R in the plasma membrane fraction of the OFC among 4 and 7 weeks of age of wild type (brown columns) and S286L-TG (green columns) were indicated, respectively. Ordinates indicate the mean ± SD (*n* = 6) of the relative expression of P2X7R per GAPDH, and abscissas indicate ages (weeks). * *p* < 0.05, relative to P2X7R expression of 4-week-old S286L, @@ *p* < 0.01, relative to the 8-week-old wild type using two-way ANOVA with Scheffe’s post hoc test. F values were [F_genotype_(1,20) = 16.6 (*p* < 0.01), F_age_(1,20) = 2.0 (*p* > 0.1), F_genotype×age_(1,20) = 5.4 (*p* < 0.05)]. The right side panel indicates the pseudo-gel images of P2X7R and GAPDH, obtained using capillary immunoblotting. Original Western Blotting Figures can be found in Supplementary Materials File S1.

To explore the mechanisms underlying the discrepancies in the BzATP-evoked release of ATP and L-glutamate in the OFC between the wild type and S286L-TG, P2X7R expression in the plasma membrane fractions in the OFC of the 4- and 7-week-old wild type and S286L-TG was analyzed. At 4 weeks old, the P2X7R expressions in the wild type and S286L-TG were almost equal; however, at 7 weeks old, the P2X7R expressions of wild type were almost equal to those of 4-week-old wild type, whereas that of 7-week-old S286L-TG was increased in comparison to 4-week-old S286L-TG and 7-week-old wild type (Figure 5). Therefore, the enhanced BzATP-evoked release of ATP and L-glutamate in the OFC is probably, at least partially, caused by the increased expression of P2X7R.

### 3.2. Purinergic Transmission in the Cultured Astrocytes

#### 3.2.1. Concentration-Dependent Effects of Long-Term Exposure to BzATP on BzATP-Evoked ATP Release from Cultured Astrocytes

P2X7R is expressed at the highest density in the microglia and neurons [76,77], but it is also expressed in astrocytes, as confirmed by mRNA and protein measurements, while connexin 43 is predominantly expressed in astrocytes [2,78,79]. In the above study, basal extracellular ATP levels in the OFC of the S286L-TG were increased compared to those of the wild type. These results suggest the possibility that astrocytes in the OFC of S286L-TG were continuously exposed to higher levels of extracellular ATP for a long time through increased P2X7R. Therefore, to explore the mechanisms of the enhanced BzATP-evoked releases of ATP and L-glutamate in the OFC of S286L-TG, the effects of exposure to BzATP for 14 d (DIV14-28) on astroglial ATP release were examined in cultured astrocytes of wild type and S286L-TG.

**Figure 6:**
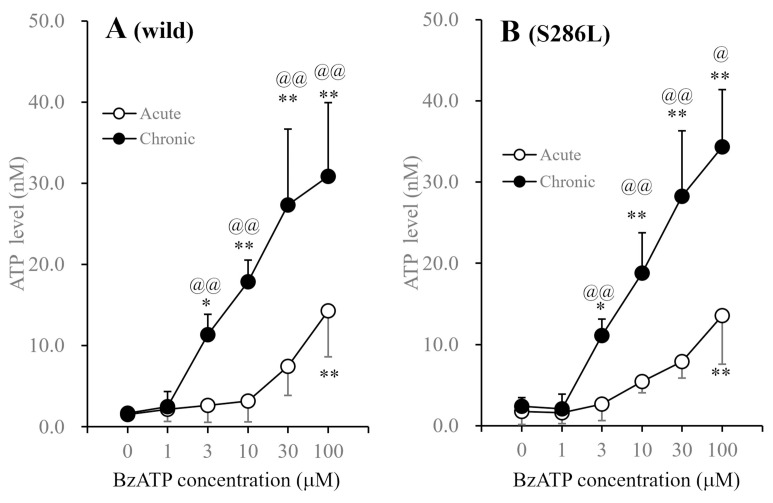
Concentration-dependent effects of BzATP on astroglial ATP release from cultured astrocytes of wild type and S286L-TG. After the incubation in fDMEM without (acute: opened circles) and with (chronic: closed circles) 1 μM BzATP for 14 d (DIV14-28), cultured astrocytes were incubated in ACSF containing BzATP (ranging from 0 to 100 μM). Panel (**A**,**B**) indicate the concentration-dependent effects of BzATP on ATP release from cultured astrocytes of wild type and S286L-TG, respectively. The ordinate and abscissa indicate the mean ± SD (*n* = 6) of extracellular ATP levels during BzATP-evoked stimulation and BzATP concentration (μM), respectively. * *p* < 0.05, ** *p* < 0.01 relative to BzATP free (0), @ *p* < 0.05, @@ *p* < 0.01 relative to acute using two-way ANOVA with Scheffe’s post hoc test. F values in concentration dependence in the wild type and S286L-TG were [F_exposure_(1,60) = 88.4 (*p* < 0.01), F_BzATP_(5,60) = 40.9 (*p* < 0.01), F_exposure×BzATP_(5,60) = 10.4 (*p* < 0.01)] and [F_exposure_(1,60) = 124.0 (*p* < 0.01), F_BzATP_(5,60) = 57.9 (*p* < 0.01), F_exposure×BzATP_(5,60) = 14.9 (*p* < 0.01)], respectively.

After incubation in fDMEM without BzATP for 14 d, cultured astrocytes were incubated in ACSF containing BzATP (concentration: 1–100 μM) for 20 min. Exposure to BzATP increased the degree of ATP release from astrocytes in both the wild type and S286L-TG in a concentration-dependent manner; however, a significant increase in astroglial ATP release required >100 μM BzATP (Figure 6).

After incubation in fDMEM containing 1 µM BzATP for 14 d (DIV14-28), which was ineffective in acutely inducing astroglial ATP release, exposure to BzATP also increased ATP release from the astrocytes of both wild type and S286L-TG in a concentration-dependent manner. Long-term exposure to 1 μM BzATP quantitatively enhanced BzATP-evoked ATP release and sensitivity to BzATP, as the threshold levels of BzATP for astroglial ATP release from astrocytes incubated in fDMEM with or without 1 μM BzATP were 100 μM and 3 μM, respectively (Figure 6). Notably, no differences were observed between wild-type and S286L-TG astrocytes with regard to the effect of BzATP on the release of astroglial ATP release.

#### 3.2.2. Effects of JNJ47965567 and Gap19 on BzATP-Evoked Astroglial ATP Releases

In the above study, differences in the responses to short-term (20 min) and long-term (14 d) exposures to BzATP between astrocytes of wild type and S286L-TG were not observed. Thus, to clarify the mechanisms of the stimulatory effects of short-term and long-term exposures to BzATP on astroglial ATP release, the effects of 1 μM JNJ47965567 and 20 μM Gap19 (selective astroglial hemichannel containing connexin 43) on BzATP-evoked astroglial ATP release were determined using cultured astrocytes from the wild type.

**Figure 7:**
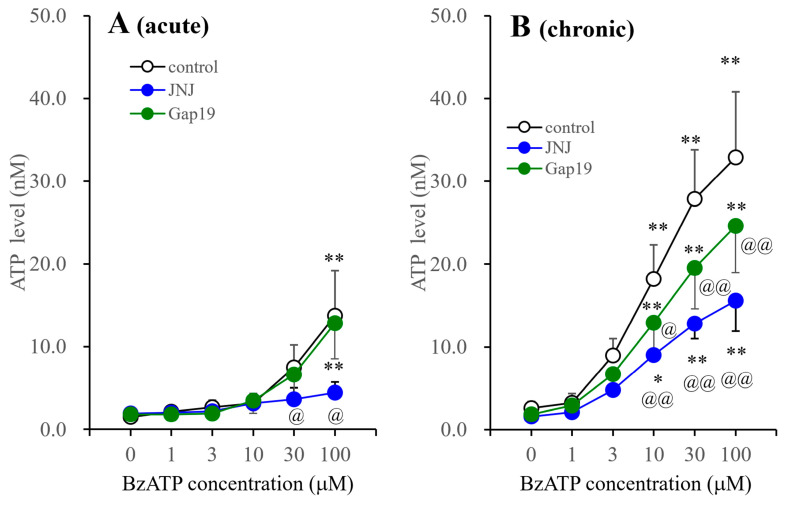
Effects of JNJ47965567 and Gap19 on BzATP-evoked astroglial ATP release from cultured astrocytes. Effects of 1 μM JNJ47965567 and 20 μM Gap19 (selective connexin 43 containing astroglial inhibitor) on BzATP-evoked ATP release from cultured astrocytes incubated in fDMEM without (acute) and with (chronic) 1 μM BzATP for 14 d (DIV14-28). After the washout, cultured astrocytes were incubated in ACSF containing BzATP (ranging from 0 to 100 μM) without (control: open circles) or with 1 μM JNJ47965567 (blue circles) or 20 μM Gap19 (green circles). Panel (**A**,**B**) indicate the concentration-dependent effects of BzATP on ATP release from cultured astrocytes incubated in fDMEM without (acute) and with (chronic) 1 μM BzATP for 14 d, respectively. The ordinate and abscissa indicate the mean ± SD (*n* = 6) of extracellular ATP levels during BzATP-evoked stimulation and BzATP concentration (μM), respectively. * *p* < 0.05, ** *p* < 0.01 relative to BzATP free (0), @ *p* < 0.05, @@ *p* < 0.01 relative to the control using two-way ANOVA with Scheffe’s post hoc test. F values in acutely and chronically administered astrocytes were [F_agent_(2,90) = 12.3 (*p* < 0.01), F_BzATP_(5,90) = 48.6 (*p* < 0.01), F_agent×BzATP_(10,90) = 6.4 (*p* < 0.01)] and [F_agent_(2,90) = 49.5 (*p* < 0.01), F_BzATP_(5,90) = 133.7 (*p* < 0.01), F_agent×BzATP_(10,90) = 6.5 (*p* < 0.01)], respectively.

JNJ47965567 suppressed the BzATP-evoked ATP release of astrocytes incubated in fDMEM with or without BzATP for 14 d (DIV14-28) (Figure 7). The inhibitory effects of JNJ47965567 on the BzATP-evoked ATP release from astrocytes incubated in BzATP-free fDMEM were observed at concentrations of >30 μM, whereas those from astrocytes incubated in fDMEM containing 1 μM BzATP were observed at concentrations of >10 μM. Gap19 (20 μM) did not affect the BzATP-evoked ATP releases from astrocytes incubated in BzATP-free fDMEM, but suppressed the release from astrocytes incubated in fDMEM containing 1 μM BzATP (Figure 7).

#### 3.2.3. Effects of BzATP, FR180204 and 10-DEBC on the Expressions of P2X7R, Connexin 43, pAkt and pErk in Cultured Astrocytes

To explore the mechanisms underlying the stimulatory effects of BzATP on astroglial ATP release, the expressions of P2X7R and connexin 43 in the plasma membrane fraction of cultured astrocytes were determined. It has already been demonstrated that the intracellular signaling of Akt and Erk play important roles in increasing connexin 43 expression on the plasma membrane in S286L-TG via the activation of connexin 43 trafficking [2,41,47]. Based on previous findings, we sought to explore the increased expression of P2X7R in the OFC of S286L-TG and the effects of the interaction between BzATP and inhibitors of P2X7R, Akt and Erk on the expression of P2X7R in plasma membrane fractions of cultured astrocytes. The activation of P2X7R enhances the signaling of both Akt and Erk, and enhanced Akt signaling increases the expressions of P2X7R and connexin 43 [41,47,80]. Based on these previous findings, the effects of long-term exposure to 1 μM BzATP, 20 μM FR180204 (Erk inhibitor) and 10 μM 10-DEBC (Akt inhibitor) for 14 d (DIV14-28) on the expressions of P2X7R and connexin 43 in the plasma membrane fractions, and the expressions of pAkt and pErk in the total lysate of cultured astrocytes, were determined.

**Figure 8:**
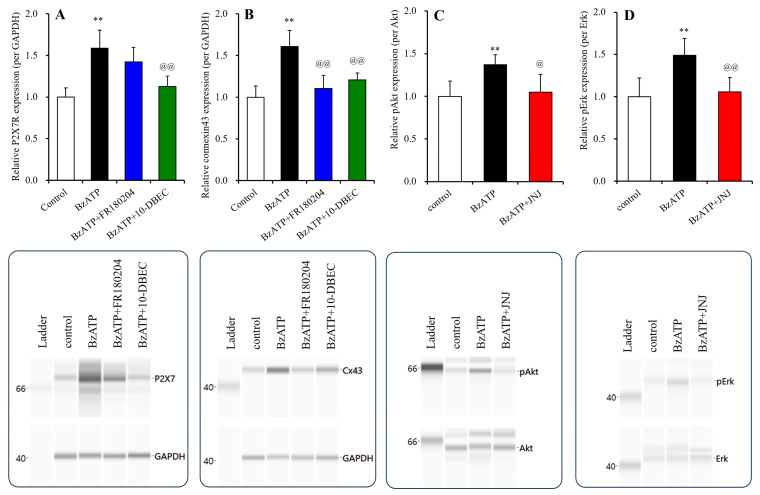
Effects of long-term exposure to BzATP on the expression of P2X7R and connexin 43 and signaling of Akt and Erk in the cultured astrocytes. Interactions among long-term (for 14 d: DIV14-28) exposures to 1 μM BzATP, 10 μM 10-DEBC (Akt inhibitor) and 20 μM FR180204 (Erk inhibitor) on the expression of P2X7R and connexin 43 in the plasma membrane fractions of cultured astrocytes are indicated in panels (**A**,**B**), respectively. The effects of chronic exposure to 1 μM BzATP on pAkt and pErk are indicated in panels (**C**,**D**), respectively. Ordinates indicate the mean ± SD (*n* = 6) of the relative expression of P2X7R per GAPDH, connexin 43 per GAPDH, pAkt per Akt or pErk per Erk. ** *p* < 0.01 relative to the expression of the control, and @ *p* < 0.05, @@ *p* < 0.01, relative to long-term exposure to 1 μM BzATP alone using one-way ANOVA with Scheffe’s post hoc test. F values of the expression of P2X7R and connexin 43 were [F(3,20) = 16.6 (*p* < 0.01)] and [F(3,20) = 19.1 (*p* < 0.01)], respectively. F values of the expression of pAkt and pErk were [F(2,15) = 8.2 (*p* < 0.01)] and [F(2,15) = 10.9 (*p* < 0.01)], respectively. The lower side panels indicate the pseudo-gel images of P2X7R, connexin 43, pAkt and pErk, obtained using capillary immunoblotting. Original Western Blotting Figures can be found in Supplementary Materials File S1.

Long-term exposure to 1 μM BzATP increased the expressions of both P2X7R and connexin 43 in the plasma membrane fractions and activated pAkt and pErk (Figure 8). The increasing expression of connexin 43 was suppressed by both 10-DEBC and FR180204. In contrast, the increased expression of P2X7R was suppressed by 10-DEBC, but not by FR180204 (Figure 8).

#### 3.2.4. Time-Dependent Effects of BzATP on Astroglial ATP Releases Induced by Ripple and Fast Ripple Burst Stimulations

The accumulation of excitability in astrocytes or astroglial hemichannels induced by physiological ripple burst and epileptogenic fast ripple burst has been reported to play important roles in the development of epileptogenesis and the age-dependent onset of ADSHE seizures in S286L-TG via enhanced Akt/Erk signaling [2,41]. Indeed, long-term exposure to HFO-evoked stimulations increased connexin 43 expression in the astroglial plasma membrane via the activation of the signaling of Akt/Erk [2,41]. The results in the above studies suggest that the increases in the basal extracellular ATP levels and P2X7R expression in the OFC of S286L-TG are also probably induced by HFO-evoked stimulations. Therefore, the effects of HFO burst stimulation for 3 days (DIV25-28) [41] on astroglial ATP release and the P2X7R expression in cultured astrocytes incubated in fDMEM containing 1 μM BzATP astrocytes for 14 d (DIV14-28), were determined.

**Figure 9:**
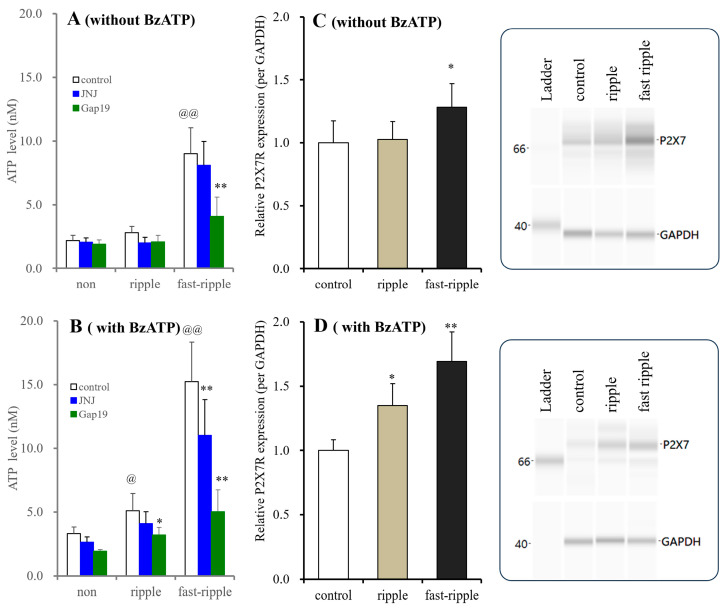
Time-dependent effects of long-term exposure to BzATP on HFO-evoked ATP release and P2X7R expression in the cultured astrocytes. Astrocytes were incubated in fDMEM without or with 1 μM BzATP for 14 d (DIV14-28). During DIV25-28, astrocytes also received HFO-evoked stimulations (ripple- or fast ripple-evoked stimulations) for 3 d. After the washout, cultured astrocytes were incubated in ACSF without (“non”) or with 1 μM JNJ47965567 (blue columns) or 20 μM Gap19 (green columns), and they then received HFO-evoked stimulation again. Panels (**A**,**B**) indicate the HFO-evoked ATP releases from cultured astrocytes incubated in fDMEM without or with 1 μM BzATP for 14 d, respectively. Ordinates indicate the mean ± SD (*n* = 6) of extracellular ATP levels during HFO-evoked stimulation (nM). @ *p* < 0.05, @@ *p* < 0.01 relative to non HFO-evoked stimulation, * *p* < 0.05, ** *p* < 0.01 relative to the control using two-way ANOVA with Scheffe’s post hoc test. F values of long-term exposure without and with 1 μM BzATP for 14 d were [F_HFO_(2,45) = 10.6 (*p* < 0.01), F_agent_(2,45) = 78.9 (*p* < 0.01), F_agent×HFO_(4,45) = 7.7 (*p* < 0.01)] and [F_HFO_(2,45) = 45.7 (*p* < 0.01), F_agent_(2,45) = 159.5 (*p* < 0.01), F_agent×HFO_(4,45) = 19.8 (*p* < 0.01)], respectively. Panels (**C**,**D**) indicate the effects of long-term exposure without and with 1 μM BzATP for 14 d (DIV14-28) with HFO-evoked simulation for 3 d (DIV25-28) on P2X7R expression in the plasma membrane fractions of cultured astrocytes. The ordinate indicates the mean ± SD (*n* = 6) of the relative expression of P2X7R per GAPDH. * *p* < 0.05, ** *p* < 0.01 relative to the expression of the control, using one-way ANOVA with Scheffe’s post hoc test. F values of long-term exposure without and with 1 μM BzATP for 14 d were [F(2,15) = 5.2 (*p* < 0.05)] and [F(2,15) = 24.6 (*p* < 0.01)], respectively. The right side panel indicates the pseudo-gel images of P2X7R, obtained using capillary immunoblotting. Original Western Blotting Figures can be found in Supplementary Materials File S1.

In astrocytes incubated in BzATP-free fDMEM, ripple-evoked stimulation did not affect astroglial ATP release, while fast ripple-evoked stimulation increased it. JNJ47965567 (1 μM) did not affect ripple-evoked or fast ripple-evoked ATP release. Gap19 (20 μM) did not affect ripple-evoked ATP release, but it decreased fast ripple-evoked ATP release (Figure 9). The expression of P2X7R was not affected by ripple-evoked stimulation and was increased by fast ripple-evoked stimulation (Figure 9).

## 4. Discussion

### 4.1. Functional Abnormality of Astroglial Purinergic Transmission in S286L-TG

The present study has demonstrated the age-dependent development of functional abnormalities in purinergic transmission in the OFC (a focus region of ADSHE seizure) of S286L-TG rats. Functional abnormalities in purinergic transmission in 4-week-old S286L-TG could not be identified. In contrast, at 7 weeks old, before the onset of ADSHE seizure but by the time interictal discharges had been displayed [2], ATP release and the expression of P2X7R were already increased in the OFC. The enhanced ATP release in 10-week-old S286L-TG (after the onset of ADSHE seizures) was almost equal to that at 7 weeks old. These results indicate the possibility that increasing ATP release and P2X7R expression in the OFC play important roles in epileptogenesis rather than in ictogenesis. However, no differences were detected between wild-type and S286L-TG astrocytes in terms of ATP release or P2X7R expression. Long-term exposure (for 14 d) to BzATP below the threshold concentration (1 μM), which could not acutely increase astroglial ATP release, enhanced the signaling of both Akt and Erk, increased the expressions of P2X7R and connexin 43, and enhanced the sensitivity to the P2X7R agonist (resulting in increasing astroglial ATP release). Therefore, astrocytes in S286L-TG probably feature no functional abnormalities associated with the Chrna4 mutation; however, long-term exposure to BzATP and/or HFO excitability contribute to enhanced purinergic transmission in the astrocytes via the upregulations in P2X7R and/or connexin 43 induced by the increased intracellular signaling of Akt/Erk. These results suggest that the long-term exposure of astrocytes to moderate levels of P2X7R agonist mimics the functional abnormalities of purinergic transmission in the ADSHE focus regions of S286L-TG, suggesting that the sustained increase in ATP release may play important roles in the development of epileptogenesis in S286L-TG.

Various studies have reported upregulated P2X7R in the brain in both experimental rodent models of epilepsy and patients with epilepsy [81,82,83]; however, whether upregulated P2X7R contributes to ictogenesis/epileptogenesis or is a consequence of epileptic seizures remains to be clarified [6,79]. P2X7R is widely expressed in the brain, including the neurons and glia [76,77]. The upregulation of P2X7R, associated with epilepsy, has been observed predominantly in neuronal presynaptic terminals [82], where it regulates neurotransmitter exocytosis [84,85]. The application of BzATP elicits increasing frequencies of spontaneous and miniature excitatory postsynaptic currents, consequently enhancing excitatory glutamatergic neurotransmission [86]. These previous findings suggest that drastic increases in P2X7R expression in the presynaptic terminals of epileptic rodents may contribute to ictogenesis. P2X7R expression in astrocytes is lower than that in the neurons, but the functions of the cation channels in astroglial P2X7R have been identified [87]. P2X7Rs contain ligand-gated cation channels, similar to other P2X receptor families. However, P2X7R shows features that are distinct from other members in the P2X receptor family. P2X7R has a low affinity for ATP and requires high levels of extracellular ATP (>100 μM) for its activation [49]. Short-term exposure (msec order) to ATP causes P2X7R to selectively permeate cations, while sustained or repetitive exposure to ATP causes P2X7R to intrinsically dilate to allow the non-selective permeation of large molecules (up to ~900 Da), including several excitatory gliotransmitters [49]. Furthermore, P2X7R possibly forms a complex with pannexin 1, eliciting CBX-sensitive gliotransmitter release [75]. These functional features of P2X7R suggest its hyperactivation is involved in the pathomechanisms of epilepsy. Taking into account previous findings, this study shows the possibility that the upregulation of astroglial P2X7R is involved in the development of epileptogenesis rather than ictogenesis, since P2X7R expression increased age-dependently in the ADSHE focus region (OFC) before ADSHE seizure onset in S286L-TG.

In addition to these established P2X7R functions, this study indicates the candidate functions of P2X7R in epileptogenesis, showing that sustained exposure to the P2X7R agonist (by 1 μM BzATP, which cannot acutely increase astroglial ATP release) increased the expression of P2X7R/connexin 43 and enhanced the signaling of Akt/Erk. The Akt/Erk signaling pathways are both downstream signaling cascades of P2X7R [88,89]. The expression of P2X7R is positively regulated by Akt but not by Erk [89,90], while the enhanced signaling of both Akt and Erk increases the astroglial connexin 43-containing hemichannel [2,41,47]. HFO bursts have been shown to activate Erk/Akt signaling, resulting in increased connexin 43 expression as a result of epileptogenesis in S286L-TG [41]. This study also demonstrated that long-term exposure to the P2X7R agonist synergistically enhanced the stimulatory effects of HFOs on Akt/Erk signaling. These results regarding the sustained modest activation of P2X7R suggest that the positive interaction between P2X7R and HFOs and their effects on the increased expressions of P2X7R and connexin 43 through the activation of Akt/Erk signaling play important roles in the development of epileptogenesis in S286L-TG.

In the present study, BzATP-evoked ATP release in the OFC of wild-type was inhibited by JNJ47965567, but was not affected by CBX. On the contrary, in S286L-TG, the increased basal ATP release and the persistently increasing releases of ATP and L-glutamate, which were CBX-sensitive and JNJ47965567-insensitive, were observed after BzATP discontinuation. However, CBX did not affect ATP release during perfusion with BzATP in either the wild type or S286L-TG. Sustained or repetitive exposure to agonists changes the features of P2X7R from cationic channels to non-selective channels permeable by large molecules [49], and it also probably facilitates the complex formation of P2X7R/pannexin 1, which also permeates large molecules and displays CBX-sensitive features [75,91]. In fact, immunoprecipitation demonstrated that P2X7R binds to pannexin 1, but until recently, no experimental results supporting the P2X7R/connexin 43 complex have been derived [75,91]. The impact of the P2X7R/pannexin 1 complex on tripartite synaptic transmission in S286L-TG could not be clarified due to the lack of selective agents; however, the potent inhibition of Gap19 upon astroglial BzATP-evoked ATP release suggests the existence of a functional positive interaction between P2X7R and connexin 43. The temporal features of the inhibitory effects of CBX and JNJ47965567 on BzATP-evoked release in the microdialysis study suggest that Ca^2+^ influx through P2X7R might also activate astroglial hemichannel opening in S286L-TG (but not in wild-type) rats.

### 4.2. Limitations

This study has some limitations. We used BzATP as the P2X7R agonist to determine the effects of P2X7R on the pathomechanisms of S286L-TG, since BzATP is a more stable and potent P2X7R agonist than ATP; however, BzATP is not selective. Therefore, the possibility that any of the responses to BzATP observed in this study were modulated by a P2X receptor other than P2X7R cannot be denied. However, JNJ47965567 (a selective P2X7R antagonist) completely inhibited the increased astroglial signaling of Akt and Erk, which was induced by sustained exposure to modest concentrations of 1 μM BzATP. The impacts of adenosinergic transmission, such as the activation of anticonvulsive A1 and proconvulsive A2A receptors by adenosine metabolized from released ATP in the synaptic cleft, are important mechanisms that cannot be ignored [48,92].

As part of the microdialysis study, to inhibit astroglial hemichannels containing connexin 43 and pannexin 1, this study conducted perfusion with CBX as a nonselective hemichannel inhibitor [55,56,93], since established selective inhibitors of hemichannels containing connexin 43 (Gap19: MW = 1161.5) and pannexin 1 (10Panx: MW = 1242.4) have a high molecular weight. The diffusion from the perfusate to the extracellular space of these peptide hemichannel inhibitors is estimated to be lower than 1% [94]. Due to the methodological limitations of microdialysis, which requires diffusion through a dialysis membrane, we had to adopt CBX rather than peptide inhibitors. In spite of these limitations, the present study identified the differences in effects between JNJ47965567 (a selective P2X7R antagonist) and CBX on BzATP-evoked astroglial ATP release, finding that JNJ47965567 inhibited the initial phase of ATP release without affecting the later persistent phase, but CBX inhibited the late persistent phase without affecting the initial phase of BzATP-evoked ATP release. These results indicate that BzATP-evoked ATP release is primarily induced by P2X7R activation and then further induced following the initiation of persistent ATP release by other factors than P2X7R. Combined with a number of previous findings regarding astrocytes cultured using Gap19, the results of our microdialysis study show that the persistent phase of BzATP-evoked ATP release in the OFC is probably, at least partially, due to ATP release through pannexin 1 channels. In other words, the results of this study also suggest that the P2X7R/pannexin 1 complex in S286L-TG could not be explored due to the lack of selective P2X7R/pannexin 1 complex inhibitors. Therefore, to identify the detailed pathomechanisms of S286L-TG, including for epileptogenesis/ictogenesis, it is necessary to study the effects of other P2X, P2Y and adenosine receptors, as well as pannexins.

## 5. Conclusions

The present study demonstrated several candidate pathomechanisms of ADSHE using S286L-TG. In our in vitro experiments using cultured astrocytes, the purinergic transmission function of astrocytes from S286L-TG did not differ from that of astrocytes from wild types [2,41,47]. Similarly to previous studies, no functional abnormalities associated with purinergic transmission in the ADSHE focus region (OFC) of 4-week-old S286L-TG were observed; however, basal and P2X7R-related ATP release was enhanced in the OFC of 7-week-old S286L-TG, which is a critical period for the onset of ADSHE seizures (ADSHE seizure onset in S286L-TG occurred at >8 weeks old) [2,39,47,59]. Furthermore, P2X7R expression on the plasma membrane in the OFC of S286L-TG was increased, similarly to connexin 43. This enhanced purinergic transmission in the OFC, featuring ATP release and P2X7R expression, was mimicked using cultured astrocyte experiments in which sustained exposure to 1 μM BzATP, which could not notably increase ATP release, enhanced the expressions of P2X7R/connexin 43, the signaling of Akt/Erk and the sensitivity to agonists that increase astroglial ATP release. Additionally, HFO-induced ATP release was also enhanced by sustained exposure to modest levels of BzATP. These functional abnormalities associated with purinergic transmission are, at least partially, involved in the development of the epileptogenesis rather than ictogenesis of S286L-TG since here, enhanced purinergic transmission in the OFC was completed before the onset of ADSHE seizure.

## Data Availability

The data that support the findings of this study are available from the corresponding author upon reasonable request. Some data may not be made available due to ethical restrictions.

## Supplementary Materials

The following supporting information can be downloaded at: https://www.mdpi.com/article/10.3390/biom14020204/s1, File S1: Original western blotting figures of Figure 5, Figure 8 and Figure 9.
