# Supplementary figures and images for: Age-Dependent Activation of Purinergic Transmission Contributes to the Development of Epileptogenesis in ADSHE Model Rats
Source: Biomolecules. 2024 Feb 8;14(2):204. doi: 10.3390/biom14020204 (PMC10886636; doi:10.3390/biom14020204)

Fig5-GAPDH

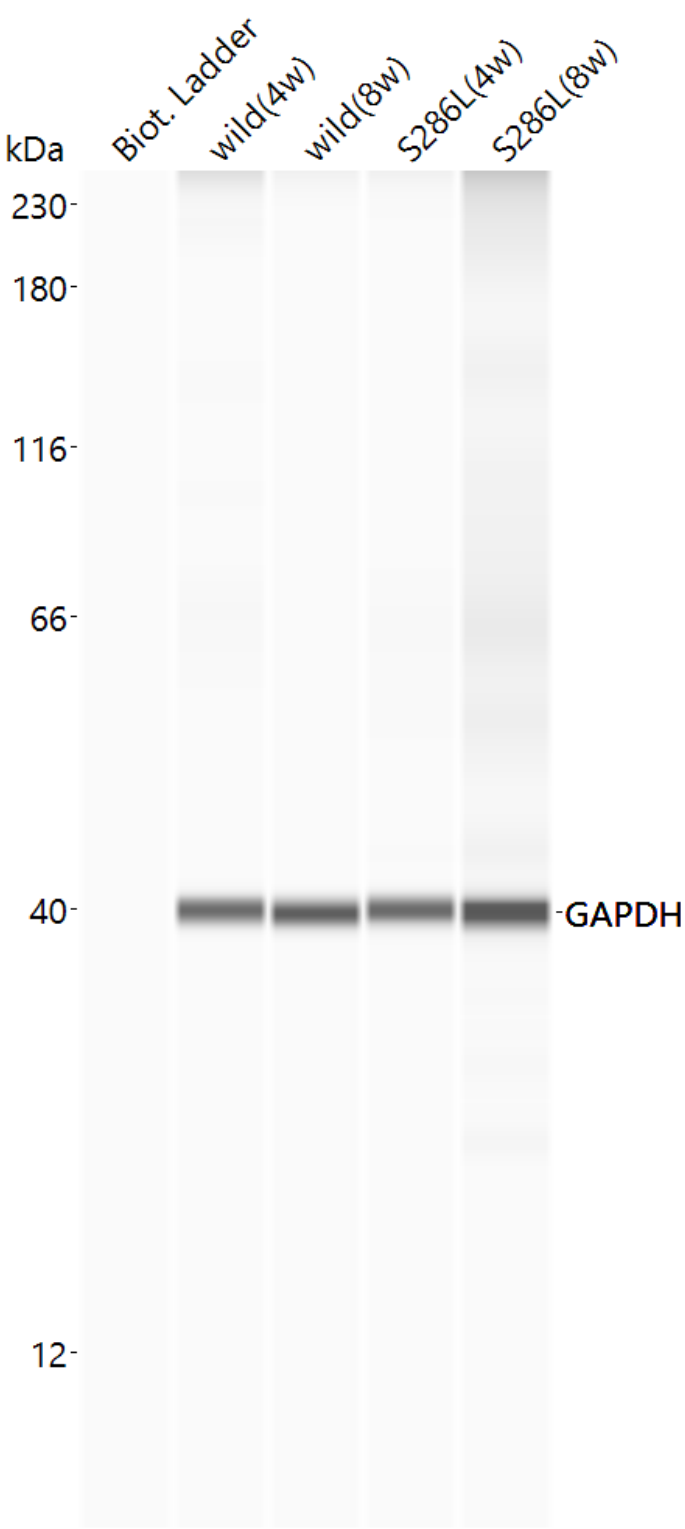

Fig5-P2X7

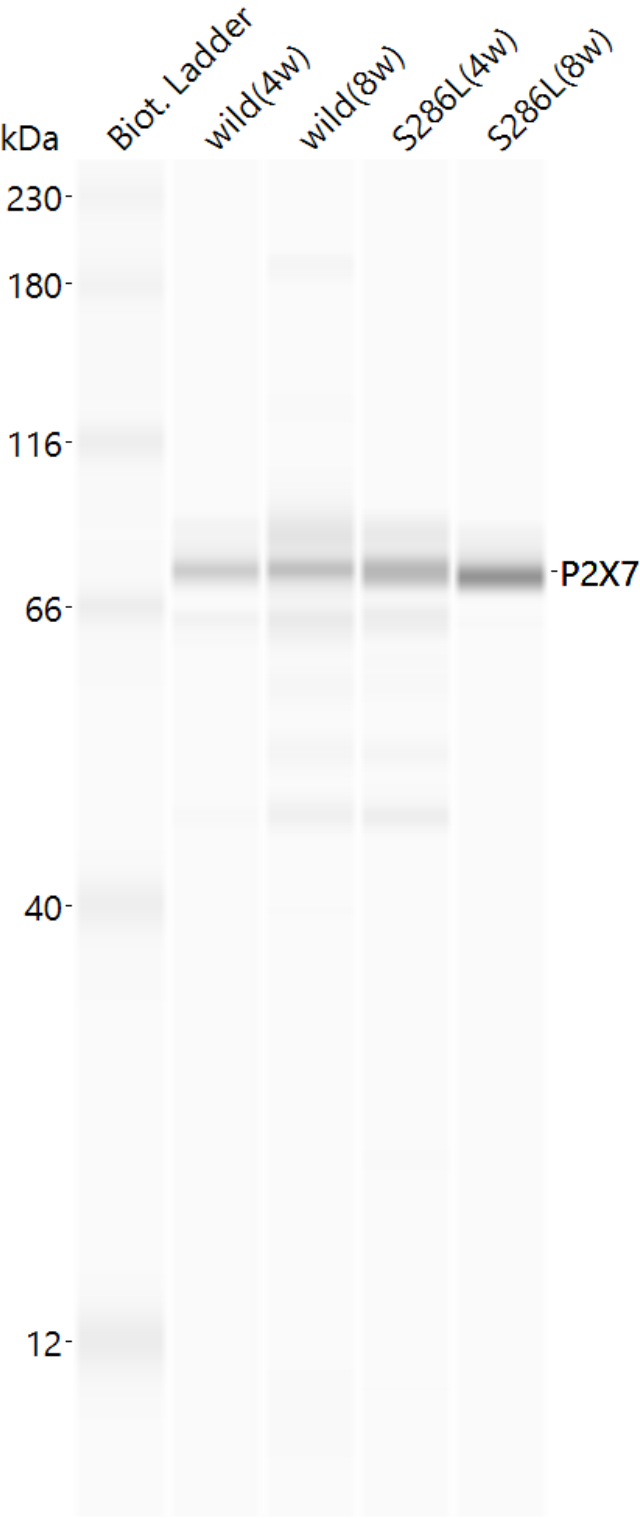

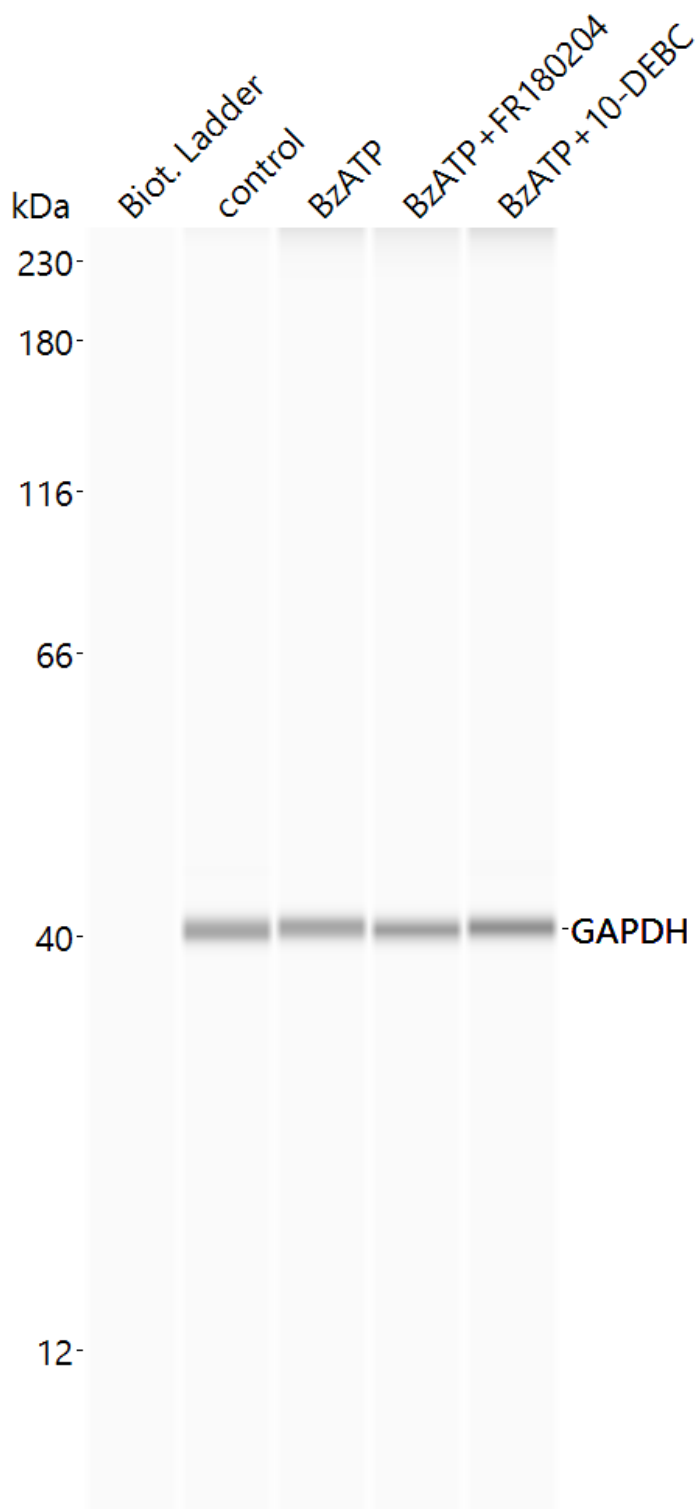

Fig8A-P2X7

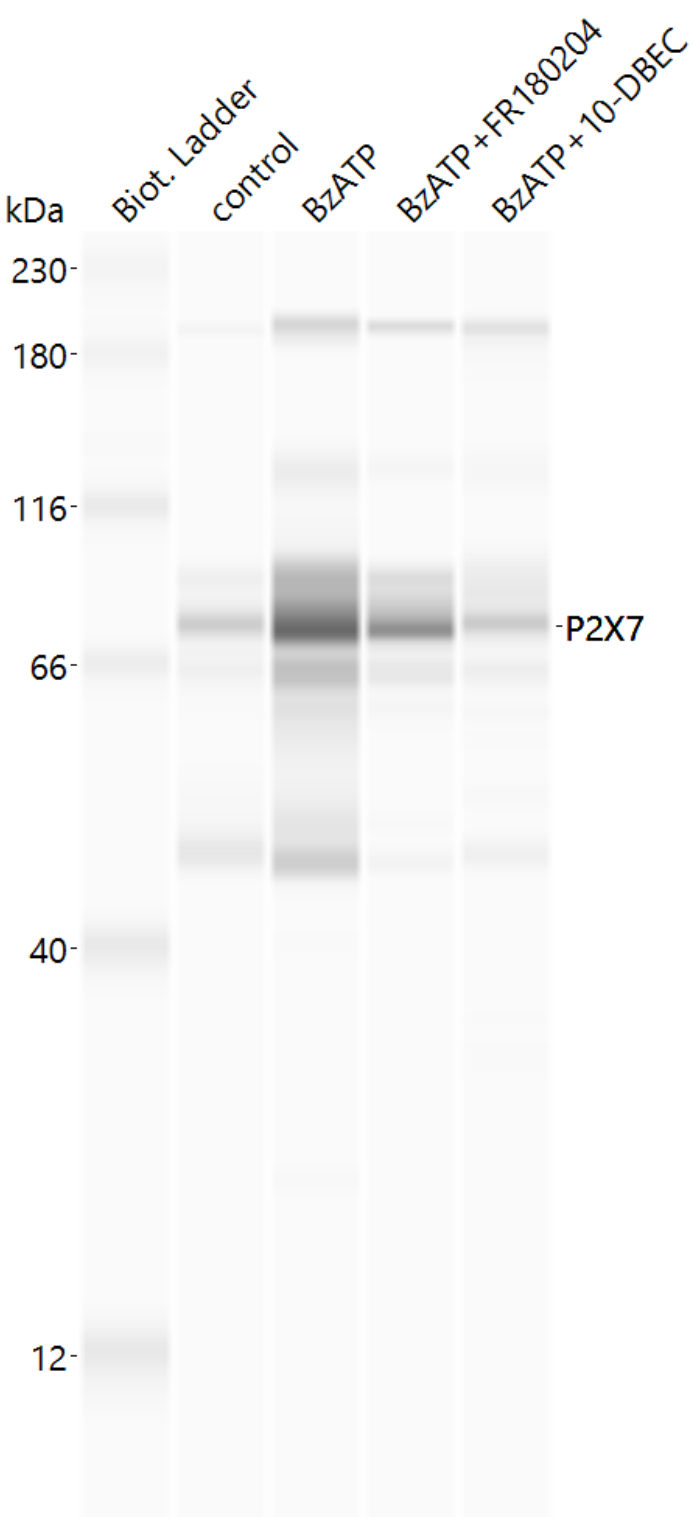

Fig8B-GAPDH

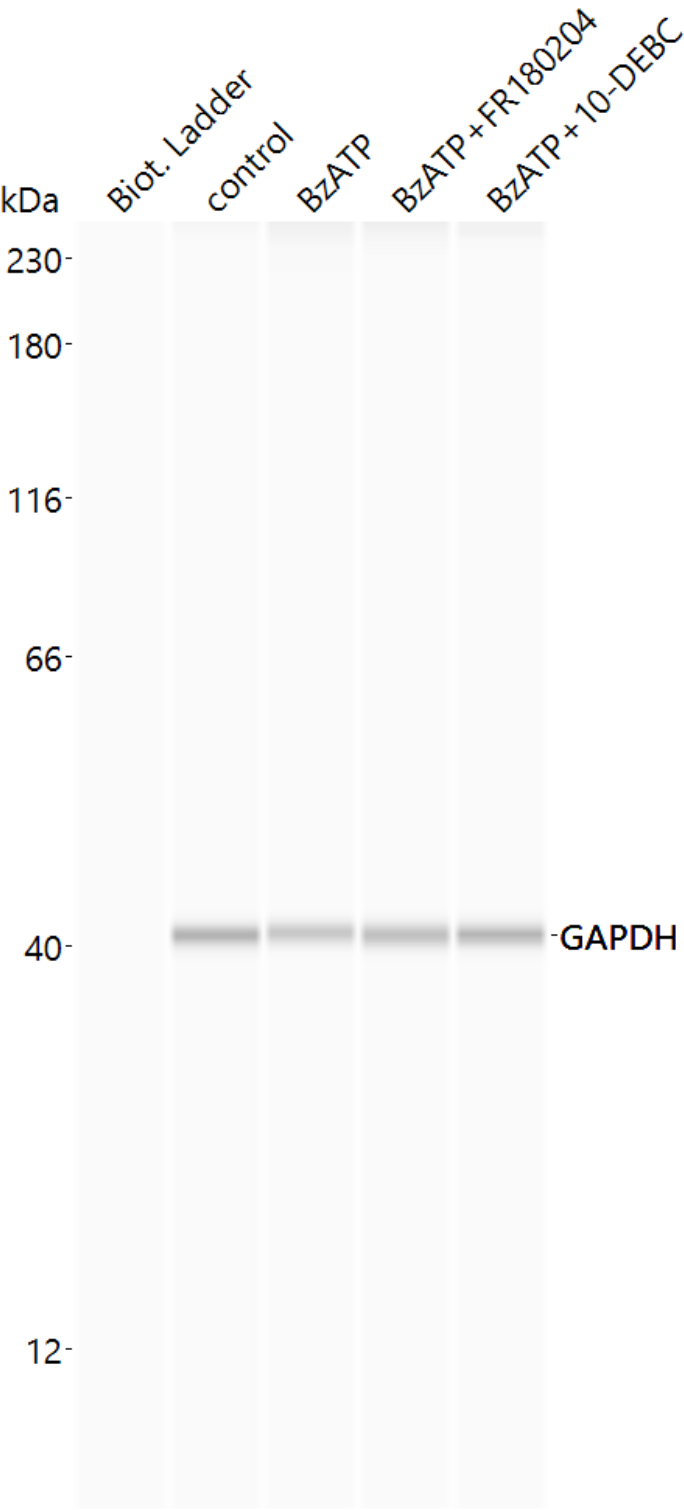

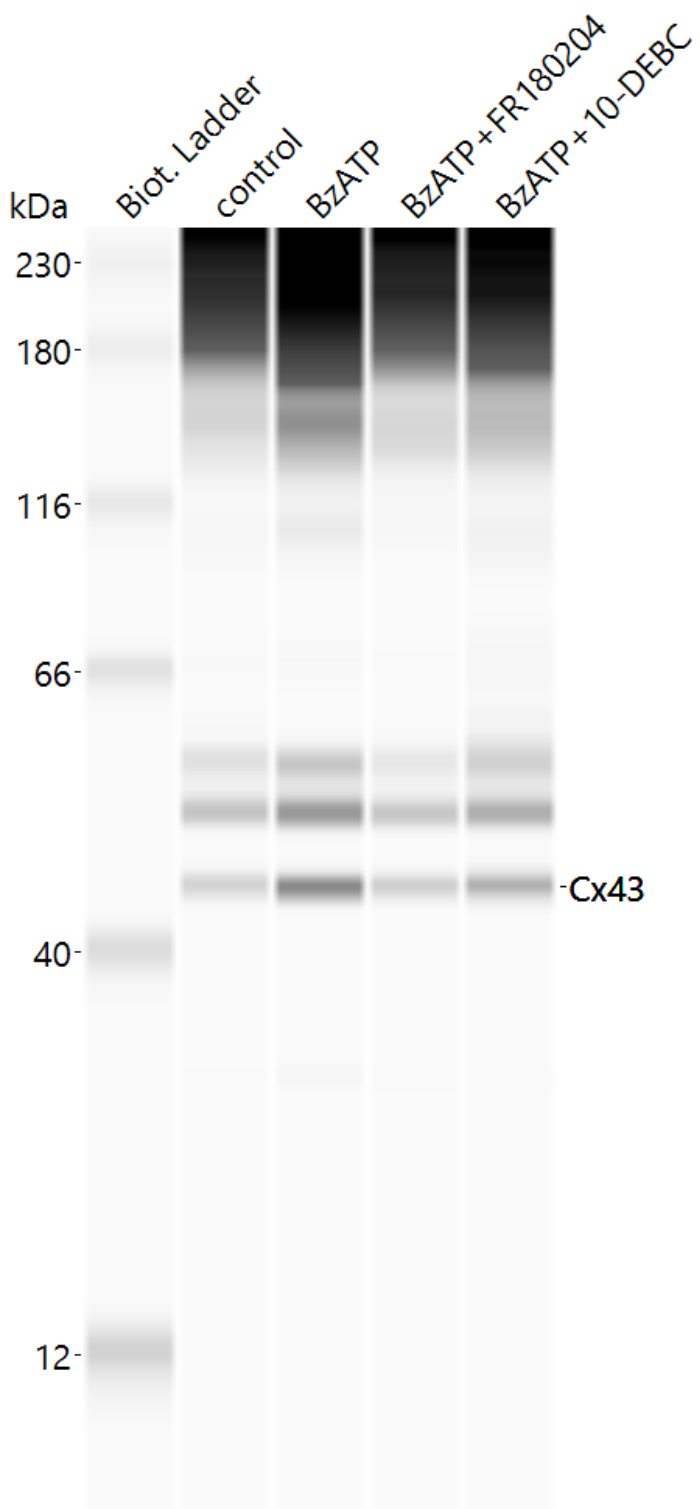

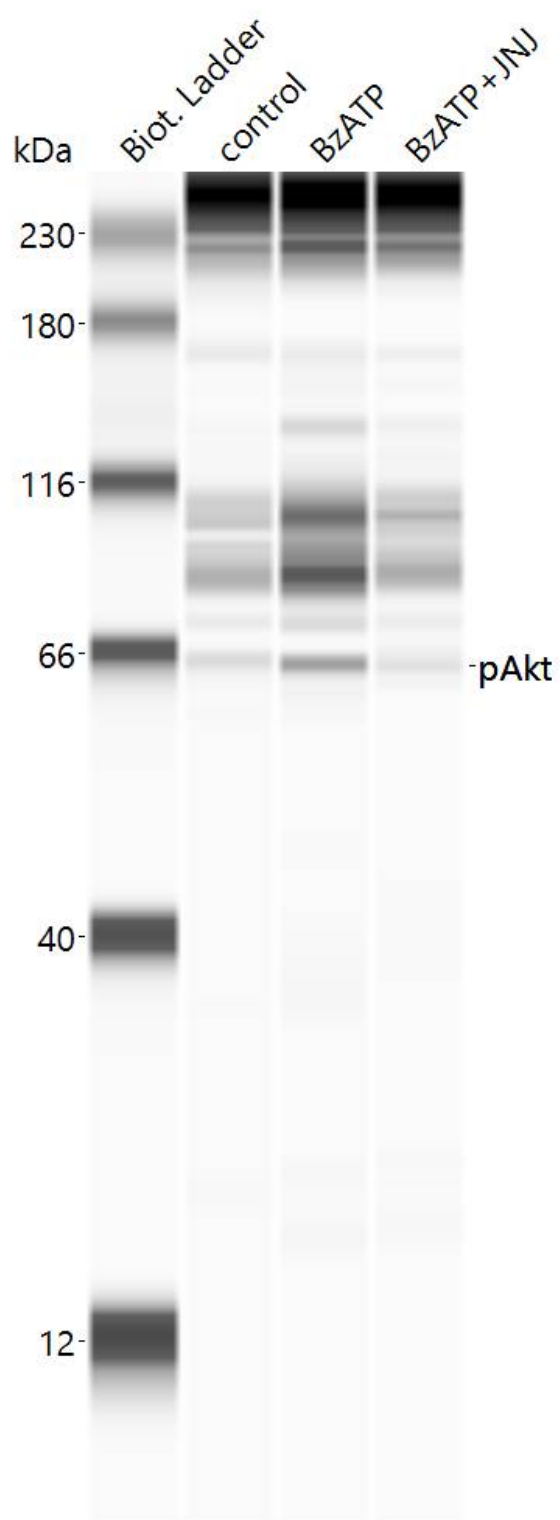

Fig8C-Akt (kinase)

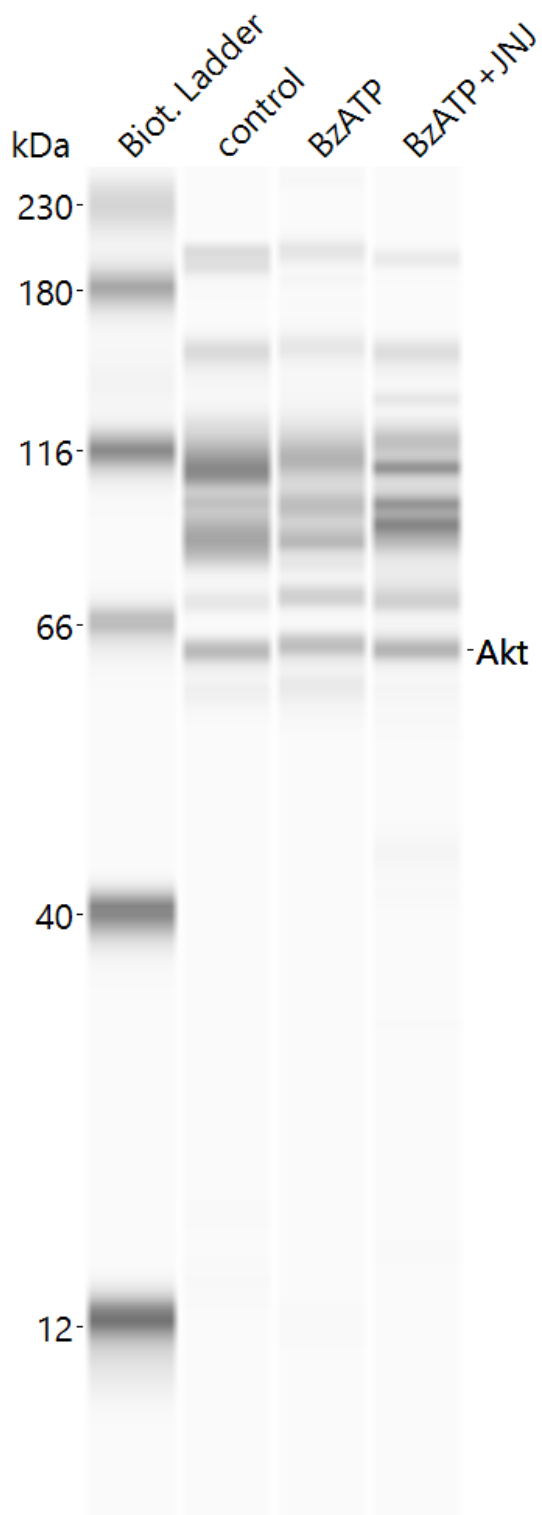

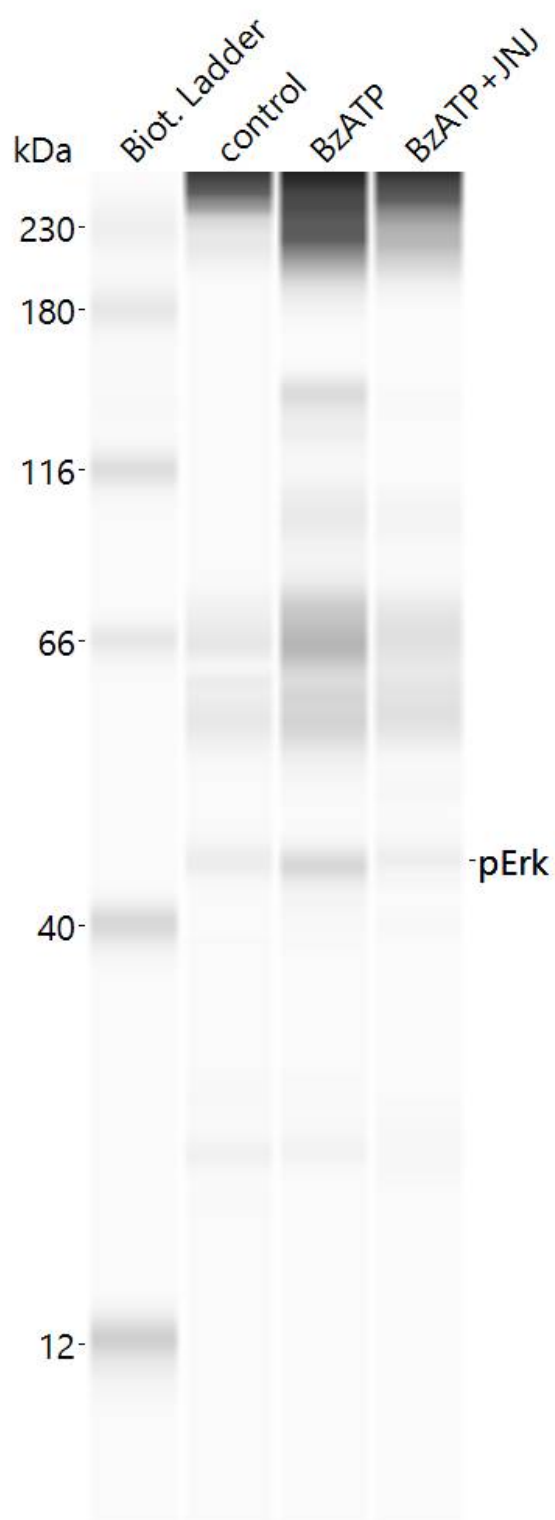

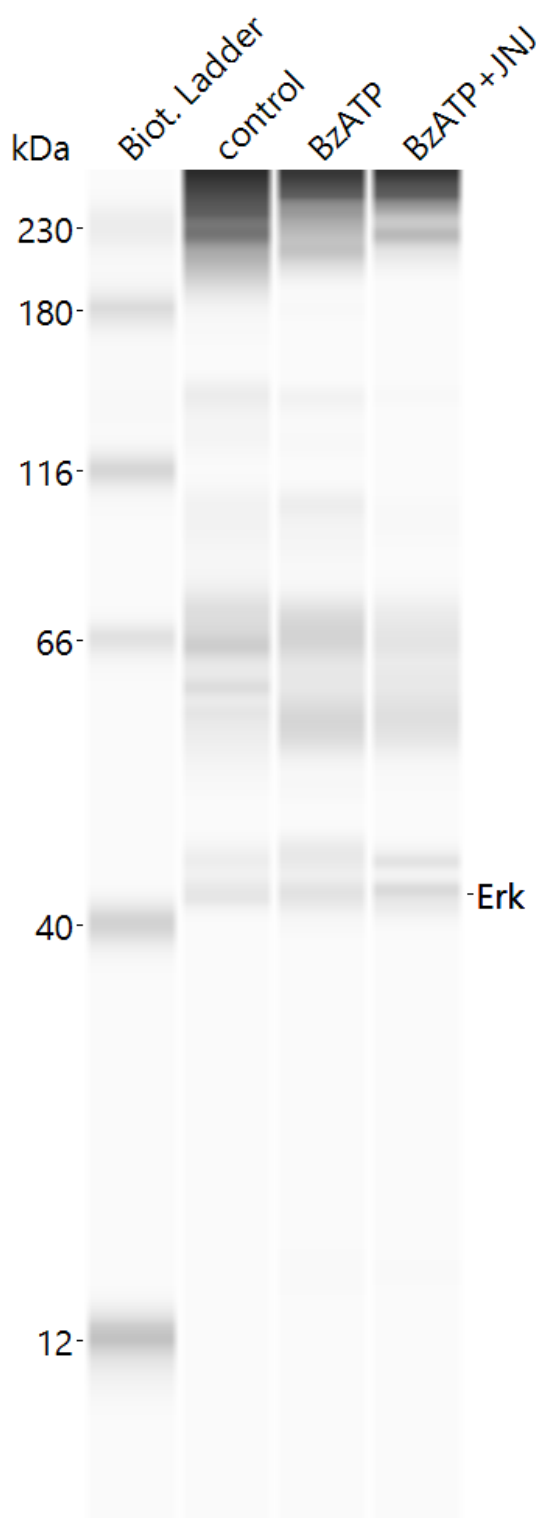

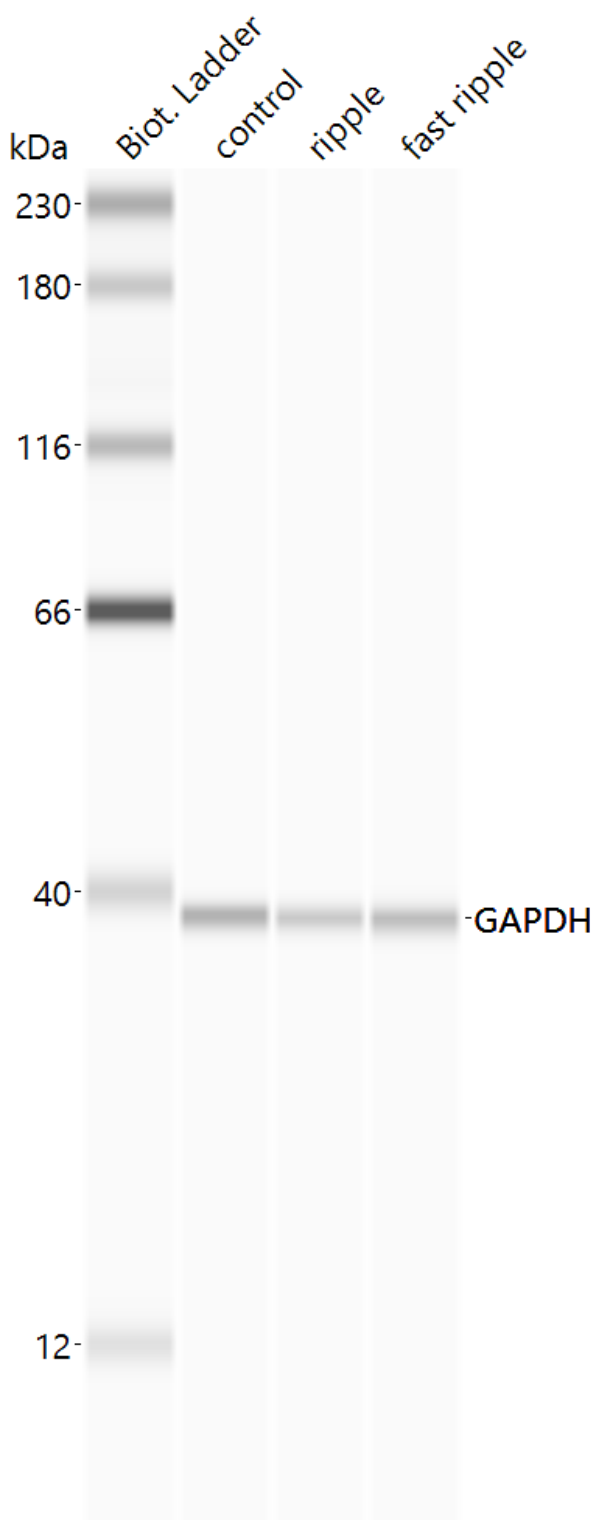

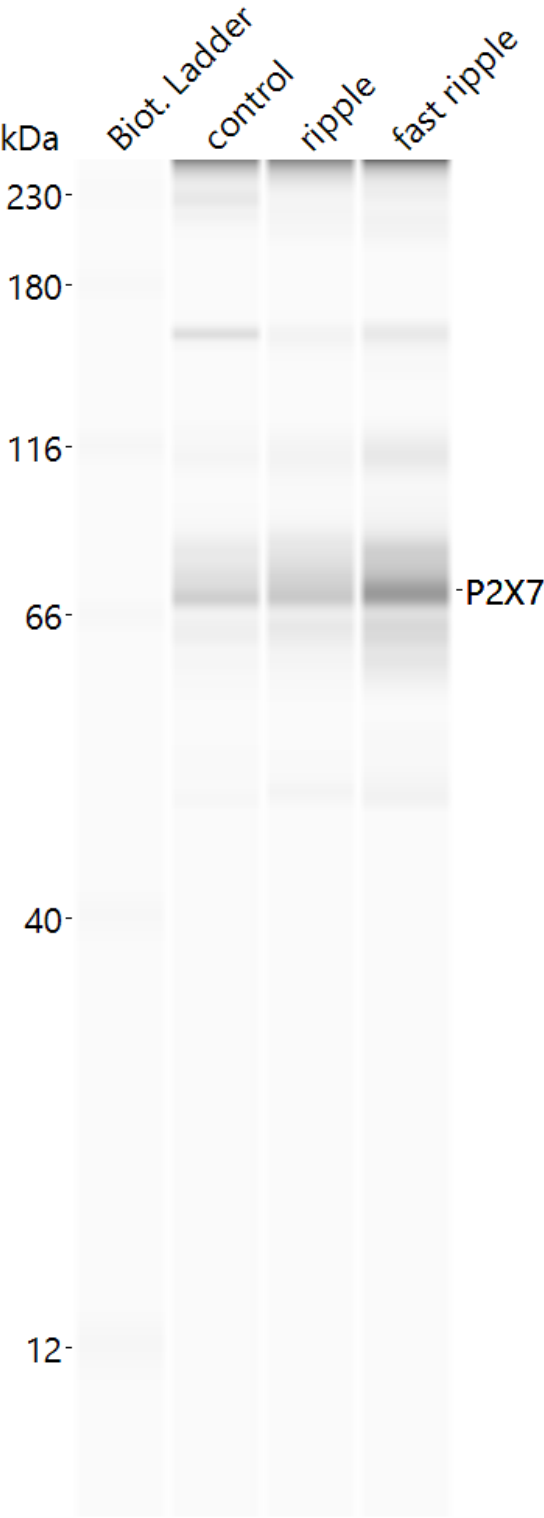

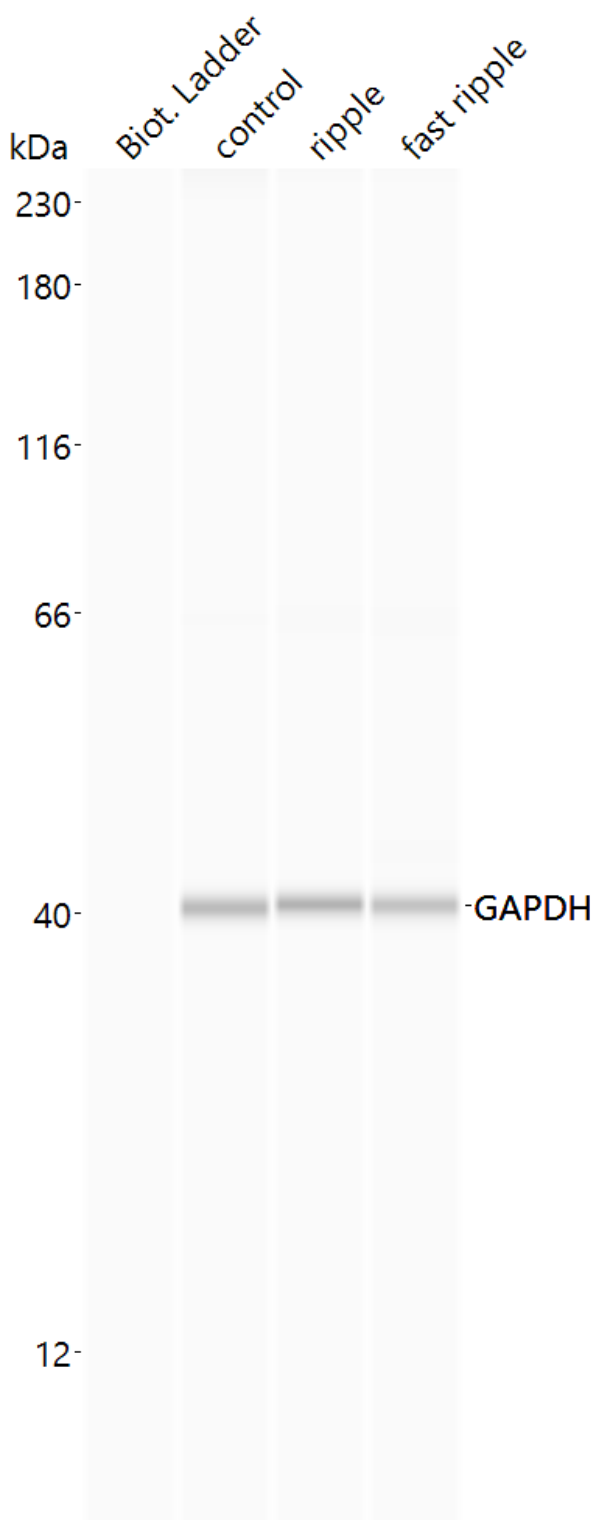

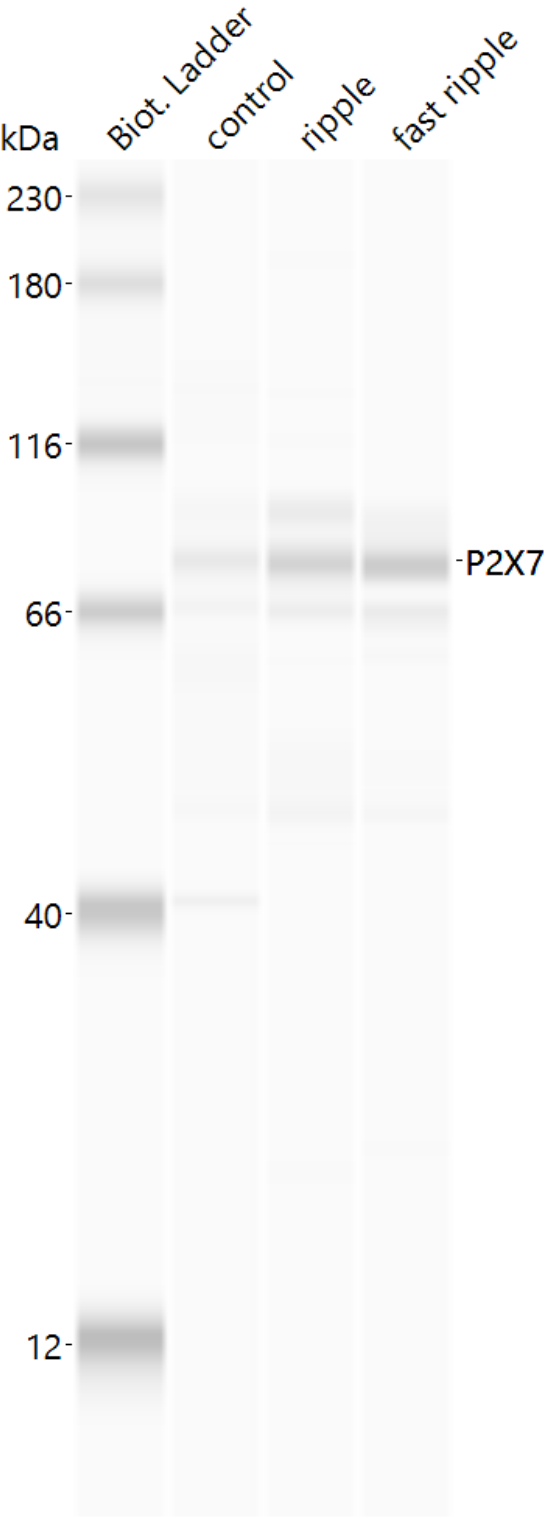

Supplement: Supplementary file 1 [file biomolecules-14-00204-s001.zip › biomolecules-2806592-supplementary.pdf]
